# Quantitation of SARS-CoV-2 neutralizing antibodies with a virus-free, authentic test

**DOI:** 10.1093/pnasnexus/pgac045

**Published:** 2022-04-14

**Authors:** Johannes Roessler, Dagmar Pich, Manuel Albanese, Paul R Wratil, Verena Krähling, Johannes C Hellmuth, Clemens Scherer, Michael von Bergwelt-Baildon, Stephan Becker, Oliver T Keppler, Alain Brisson, Reinhard Zeidler, Wolfgang Hammerschmidt

**Affiliations:** Department of Otorhinolaryngology, University Hospital, Ludwig-Maximilians-Universität München, Munich, Germany; Research Unit Gene Vectors, Helmholtz Zentrum München, German Research Center for Environmental Health, Munich, Germany; German Centre for Infection Research (DZIF), Partner site Munich, Germany; Research Unit Gene Vectors, Helmholtz Zentrum München, German Research Center for Environmental Health, Munich, Germany; German Centre for Infection Research (DZIF), Partner site Munich, Germany; German Centre for Infection Research (DZIF), Partner site Munich, Germany; Max von Pettenkofer Institute and Gene Center, Virology, National Reference Center for Retroviruses, Faculty of Medicine, Ludwig-Maximilians-Universität München, Munich, Germany; German Centre for Infection Research (DZIF), Partner site Munich, Germany; Max von Pettenkofer Institute and Gene Center, Virology, National Reference Center for Retroviruses, Faculty of Medicine, Ludwig-Maximilians-Universität München, Munich, Germany; Institute of Virology, Faculty of Medicine, Philipps-Universität Marburg, Marburg, Germany; German Centre for Infection Research (DZIF), Partner site Giessen-Marburg-Langen, Marburg, Germany; Department of Medicine III, University Hospital, Ludwig-Maximilians-Universität München, Munich, Germany; COVID-19 Registry of the LMU Munich (CORKUM), University Hospital, Ludwig-Maximilians-Universität München, Munich, Germany; COVID-19 Registry of the LMU Munich (CORKUM), University Hospital, Ludwig-Maximilians-Universität München, Munich, Germany; Department of Medicine I, University Hospital, Ludwig-Maximilians-Universität München, Munich, Germany; Department of Medicine III, University Hospital, Ludwig-Maximilians-Universität München, Munich, Germany; COVID-19 Registry of the LMU Munich (CORKUM), University Hospital, Ludwig-Maximilians-Universität München, Munich, Germany; German Cancer Consortium (DKTK), Munich, Germany; Institute of Virology, Faculty of Medicine, Philipps-Universität Marburg, Marburg, Germany; German Centre for Infection Research (DZIF), Partner site Giessen-Marburg-Langen, Marburg, Germany; German Centre for Infection Research (DZIF), Partner site Munich, Germany; Max von Pettenkofer Institute and Gene Center, Virology, National Reference Center for Retroviruses, Faculty of Medicine, Ludwig-Maximilians-Universität München, Munich, Germany; COVID-19 Registry of the LMU Munich (CORKUM), University Hospital, Ludwig-Maximilians-Universität München, Munich, Germany; UMR-CBMN CNRS-University of Bordeaux-INP, Pessac, France; Department of Otorhinolaryngology, University Hospital, Ludwig-Maximilians-Universität München, Munich, Germany; Research Unit Gene Vectors, Helmholtz Zentrum München, German Research Center for Environmental Health, Munich, Germany; German Centre for Infection Research (DZIF), Partner site Munich, Germany; Research Unit Gene Vectors, Helmholtz Zentrum München, German Research Center for Environmental Health, Munich, Germany; German Centre for Infection Research (DZIF), Partner site Munich, Germany

**Keywords:** SARS-CoV-2, virus-like particle, virus neutralization test, diagnostics, Omicron

## Abstract

Neutralizing antibodies (NAbs), and their concentration in sera of convalescents and vaccinees are a correlate of protection from COVID-19. The antibody concentrations in clinical samples that neutralize SARS-CoV-2 are difficult and very cumbersome to assess with conventional virus neutralization tests (cVNTs), which require work with the infectious virus and biosafety level 3 containment precautions. Alternative virus neutralization tests (VNTs) currently in use are mostly surrogate tests based on direct or competitive enzyme immunoassays or use viral vectors with the spike protein as the single structural component of SARS-CoV-2. To overcome these obstacles, we developed a virus-free, safe and very fast (4.5 h) in vitro diagnostic test based on engineered yet authentic SARS-CoV-2 virus-like particles (VLPs). They share all features of the original SARS-CoV-2 but lack the viral RNA genome, and thus are noninfectious. NAbs induced by infection or vaccination, but also potentially neutralizing monoclonal antibodies can be reliably quantified and assessed with ease and within hours with our test, because they interfere and block the ACE2-mediated uptake of VLPs by recipient cells. Results from the VLP neutralization test (VLPNT) showed excellent specificity and sensitivity and correlated very well with a cVNT using fully infectious SARS-CoV-2. The results also demonstrated the reduced neutralizing capacity of COVID-19 vaccinee sera against variants of concern of SARS-CoV-2 including omicron B.1.1.529, BA.1.

Significance Statement The current pandemic by SARS-CoV-2 is a major challenge to COVID-19 patients, medical staff, healthcare systems, and the general public, but also for virologists and clinical laboratories. A particular challenge are safety issues which require biological safety level 3 to work with and study the pathogen. As an alternative, we engineered VLPs, which are close-to-perfect mimics of SARS-CoV-2, are authentic in terms of viral structure and functions but are harmless bioproducts in nature. High concentrations of NAbs correlate with protection from COVID-19; thus, practical VNTs are urgently needed. We used SARS-CoV-2 VLPs in a virus-free, thus safe in vitro VNT to screen NAbs in a standardized assay format in less than 5 h.

## Introduction

In December 2019, a novel respiratory infectious disease that led to an outbreak of severe cases of pneumonia (1) marked the beginning of the ongoing pandemic caused by the severe acute respiratory syndrome coronavirus 2 (SARS-CoV-2), a virulent member of the *Coronaviridae* family. The virus likely originated from a wildlife reservoir in bats but spreads easily among humans via droplets and aerosols. As of March 2022, the associated coronavirus disease 2019 (COVID-19) accounts for more than 6 million deaths worldwide (WHO dashboard, https://covid19.who.int). Aside from subclinical infections, COVID-19 can vary from weak symptoms to mild or severe pneumonia with dyspnea, to critical clinical courses with acute respiratory distress syndrome (ARDS) requiring external ventilation and intensive care.

SARS-CoV-2 is an enveloped *Betacoronavirus* with a positive-sense single-stranded RNA genome of almost 30 kb encoding a replicase polyprotein (ORF1a/ORF1b), four structural proteins spike (S), envelope (E), membrane (M), and nucleoprotein (N, also known as nucleocapsid) and seven accessory proteins (2, 3). In detail, S is a class I fusion protein (FP), which assembles in homotrimers, comprising three S1 domains on top of three S2 units, each separated by a S1/S2 furin cleavage site. While S1 contains the receptor binding domain (RBD), S2 bears the fusion peptide and two heptad repeats, mediating membrane proximity and fusion (4). Proteolytic processing at the S1/S2 site, which often occurs during egress from a virus producing cell primes S for an additional second cleavage at the S2’ site within the S2 domain. The second cleavage facilitates presentation of the fusion peptide and uptake by the susceptible host cell (5, 6).

Angiotensin-converting enzyme 2 (ACE2) on target cells serves as cellular receptor for SARS-CoV-2, which attaches to ACE2 via the receptor binding motif (RBM) in the S1 subunit. To make the RBM accessible, S in its prefusion conformation undergoes conformational changes, exposing one RBD of the protomer in the ACE2-accessible “up” orientation (3). Receptor binding then triggers fusion at the plasma membrane or endocytosis after proteolytic processing at the S2’ site by either the cell surface protease TMPRSS2 (7) or endosomal cathepsin L (CTSL) (5) to promote membrane insertion of the fusion peptide. Subsequent cytoplasmic release of the viral RNA cargo initiates translation and further steps downstream to turn the cell into a virus factory (3).

Besides the innate immune system, adaptive immunity grants protection against SARS-CoV-2 in the form of anti-S antibodies (2) causing direct neutralization of virions as well as Fc mediated antibody-dependent cellular phagocytosis (ADCP), complement-dependent cytotoxicity (CDC), and antibody-dependent cellular cytotoxicity (ADCC) (8). The cellular immune response also supports broad and durable immune protection by CD8^+^ T cells targeting the nucleoprotein, but also with spike specific CD4^+^ T cells (9, 10).

In the course of the pandemic, several mutations in the spike gene have led to enhanced viral infectivity and spread. The first major mutation was the single amino acid mutation D614G, which caused higher viral loads and worldwide spread of the B.1 lineage (S: D614G), displacing the original Wuhan-2019 strain (11). Meanwhile, other strains such as B.1.1.7, the Alpha variant of concern (VOC) also led to enhanced transmissibility (12), which is probably due to the N501Y mutation in S, enhancing ACE2 affinity (13). Subsequently, the Delta-VOC B.1.617.2 evolved, which was more transmissible (14, 15) and posed a twofold higher risk to become hospitalized (16). Recently the Omicron-VOC, B.1.1.529, and BA.1, emerged with more than 30 substitutions, six deletions, and three insertions in the S protein in November 2021 and rapidly became the predominant variant (17, 18).

As a result of the global effort in COVID-19 vaccine development, two mRNA vaccines, BNT162b2 by BioNTech/Pfizer and mRNA-1273 by Moderna, as well as a chimpanzee adenovector ChAdOx1 vaccine (AZD1222) by AstraZeneca were licensed by the FDA and EMA (among other vaccines). For infections with the Alpha- and Delta-VOCs, these vaccines significantly reduce the risk for symptomatic COVID-19, effectively attenuate disease severity and reduce the rate of mortality (16, 19, 20). The current vaccines, however, do not confer sterile immunity as break-through infections (BTI) even in fully vaccinated individuals can occur (21, 22). A third dose or “booster” of COVID-19 vaccines further reduced the risk for BTI and severe illness and, therefore, entered the vaccination schemes (23). Vaccine efficacy was maintained at lower level against Omicron (24), yet the variant marked the emergence of an independent SARS-CoV-2 serotype characterized by immune escape and reduced cross-neutralization of antibodies induced by previous variants (25).

Neutralizing antibodies (NAbs), induced by infection or vaccination or applied in the form of monoclonal antibodies (mAbs) or convalescent plasma (26) have the potential to establish immunity to SARS-CoV-2 and to protect from severe COVID-19. As such, the concentration of NAbs is generally accepted as a relevant correlate of protection. In line, high NAb titers were shown to be directly associated with a lower risk of symptomatic SARS-CoV-2 infections and, therefore, are highly predictive to protect from COVID-19 (27–29). Noteworthy, also anti-S IgG and anti-RBD IgG antibodies, which do not necessarily inhibit viral infection in vitro, showed acceptable correlation with protection, yet NAb titers determined with a conventional virus neutralization test (cVNT) were found to correlate best (28).

Reliable quantification of NAbs in clinical samples is problematic for several reasons. One reason lies in the diversity of VNTs and their standardization and validation (30). Undisputed “gold standard” for quantitating NAbs are cVNTs. They rely on replication competent virus stocks, which, as such, guarantee correct virion composition and a genuine infection process. Different versions of cVNTs are in use (31): (i) in multicycle assays, NAbs interfere with viral infection and replication as monitored by the amount of viral antigen generated within a defined period of time postinfection. (ii) cVNTs based on limiting dilution use the initial inactivation of the inoculum by NAbs and the reduction of the viral cytopathic effect (CPE) as a function of neutralizing antibody concentration. (iii) Plaque reduction neutralization tests (PRNTs) are based on single infected cells, which give rise to a single plaque, a localized CPE in monolayers of immobilized gel-embedded cells. cVNTs and especially PRNTs depend on the formation of CPEs and require visual enumeration or immunodetection of viral antigens. As a consequence, the tests are cumbersome to standardize between different laboratories. All cVNTs involve handling of infectious virus, which, in case of SARS-CoV-2 require typical containment measures of a BSL-3 facility and most tests take several days until readout. Consequently, several alternatives to measure NAb concentration have been developed.

Surrogate virus neutralization tests (sVNT), often performed in an ELISA format, do not specifically quantitate NAbs but antibodies that interfere with the RBD-ACE2 interaction. Therefore, they display rather weak correlation to cVNTs (32–34). Given the apparent limitations of sVNTs, pseudotyped virus neutralization tests (pVNTs) have been developed. They often rely on replication deficient viral vectors with spike as the only SARS-CoV-2-derived component and their read-out is based on de novo transcription and translation of a phenotypic reporter protein (31). As a consequence, pVNTs take 2 to 3 days in a BSL-2 laboratory. Only recently, a version of pVNT has been proposed, which makes use of a fast lentivirus-based transfer of an enzyme reporter (35). Although pVNTs are in wide use, the assembly, morphogenesis, structure, and composition of retro-, lenti-, or rhabdoviral vector particles differ from that of coronaviruses. The many versions and characteristics of different multi- or single-cycled cVNTs and pVNTs to analyze SARS-CoV-2 NAbs have recently been summarized by Khoury et al. (31).

Given the clinical relevance of NAbs and the problems with and limitations of the various VNTs, we developed a virus- and GMO-free diagnostic VNT that is safe and quick and quantifies SARS-CoV-2 NAbs at a level and quality comparable to a cVNT. We established a protocol to produce authentic virus-like particles (VLPs), which also encompass an activator peptide to trace them. The identity of the VLPs was thoroughly examined by cryoelectron microscopy (cryo-EM) as well as with biochemical methods to validate their biochemical, physical, and functional characteristics in comparison to infectious SARS-CoV-2. Our results document that SARS-CoV-2 VLPs enter target cells via ACE2, mediate membrane fusion, and deliver their luminal protein cargo into the cytosol, thus mimicking all steps of infection of the pathogen prior to viral transcription. Therefore, NAbs that provide protective immunity from SARS-CoV-2 also prevent “infection” with SARS-CoV-2 VLPs. Our test quantitates them and its results show a very high and convincing correlation with a cVNT using infectious virus and a set of double-blinded COVID-19 patient serum samples. Additionally, our work demonstrates that this VLP neutralization test (VLPNT) allows for the evaluation of VOCs by simply adapting the assay to the B.1.617.2 Delta- and B.1.1.529 BA.1 Omicron variants.

By meeting important requirements for quality, reproducibility, and rapidness, this test is a valuable tool for vaccine and therapeutic antibody development. Likely, the test is also suitable for high-throughput screening of viral entry inhibitors. As the test format is flexible it can be easily adapted to mutants of SARS-CoV-2 that may emerge in the future.

## Results

### Manufacturing of SARS-CoV-2 VLPs

SARS-CoV-2 VLPs, termed S^+^ VLPs, were generated by transient cotransfection of expression plasmids encoding all four structural proteins of the virus: S (Wuhan-2019 D614G B.1; B.1.617.2; and B.1.1.529 BA.1), M, N, and E in defined stoichiometry into HEK293T cells. To trace the S^+^ VLPs, a fifth expression plasmid was cotransfected to express a chimeric reporter protein consisting of the human CD63 tetraspanin protein and, at its carboxy terminus, an activator of split-nano-luciferase (CD63∼HiBiT). A total of 3 days after transfection, assembled S^+^ VLPs were present in large quantities in the cell culture medium. Further purification and concentration were optional and applied if needed to characterize the S^+^ VLPs in detail.

### Cryo-EM of SARS-CoV-2 VLPs

**Fig. 1.**
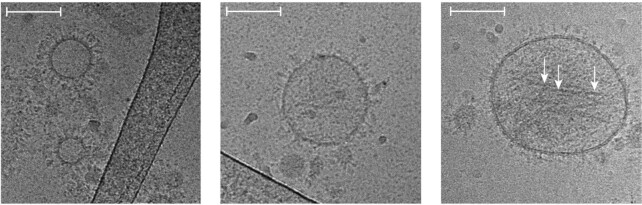
Cryo-EM images of SARS-CoV-2 VLPs (S^+^ VLPs). The images show different SARS-CoV-2 VLPs (S^+^ VLPs) of approximately 60 to 150 nm in diameter recorded by cryo-EM. The particles bear the characteristic corona of radial, dense spike-like proteins protruding from the envelopes’ intact lipid bilayer, which are characteristic for trimers of the viral glycoprotein of coronaviruses, spike (S), as observed for SARS-CoV-2 virions. The particle in the right panel shows elongated structures (white arrows), which might correspond to spike protein protrusions lying down on the vesicle surface, likely caused by surface tension effects prior to rapid freezing of the sample. White scale bars indicate 100 nm.

Cryo-EM of the S^+^ VLPs revealed spherically shaped vesicles in the range of 60 to 150 nm in diameter with a membrane consisting of an intact lipid bilayer (Fig. 1). Like SARS-CoV-2 virions (36–38), the VLPs displayed a characteristic corona of dense, needle-like radial proteins protruding perpendicularly from the membrane. On the distal ends of the protrusions spacious heads sit on slimmer stems, suggesting that these structures correspond to the viral glycoprotein spike of SARS-CoV-2. The shape and dimensions of the protrusions which are about 25 nm in length and have a stem width of 7 nm clearly support this assumption. In addition, elongated structures are observed on certain spike-bearing particles (white arrows in Fig. 1). These structures might correspond to spike protein protrusions lying down on the vesicle surface, likely caused by surface tension effects prior to plunging of the sample in cryogen. Also, evaporation might reduce the height of the liquid film causing a partial air contact of the particle's envelope and a redistribution and flattening of surface components in contrast to spikes from the periphery, which maintain their integrity in the surrounding liquid phase. Alternatively, these elongated structures may also correspond to some elements such as fibrous proteins present in the lumen of S^+^ VLPs.

SARS-CoV-2 virions contain a complex of ribonucleoproteins (N) and the ∼30 kb RNA genome, but S^+^ VLPs seemingly do not contain a similar luminal mass (Fig. 1) probably because the vRNA genome is absent. Together with N, the large vRNA molecule might act as a sizing factor, which could explain the variability in diameter seen in the S^+^ VLP preparations. Other than that, our S^+^ VLPs seem to mimic SARS-CoV-2 virions structurally (Fig. 1).

### Molecular characterization of SARS-CoV-2 VLPs (S^+^ VLPs)

Spike, S, the viral FP of SARS-CoV-2 is a highly glycosylated type I transmembrane protein, which assembles as homotrimers. It encompasses the two domains S1 and S2, which are proteolytically separated by cellular furin protease. After furin cleavage, S1 and S2 remain noncovalently associated (39), but it appears as if furin cleavage is dispensable for infection (40).

**Fig. 2.**
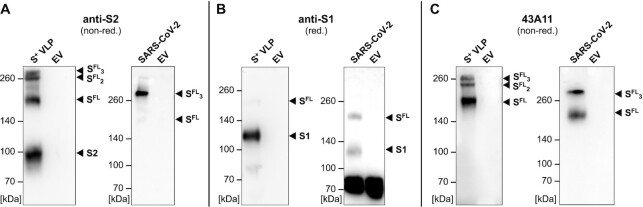
Spike WB analyses of protein lysates from S^+^ VLPs and SARS-CoV-2 virus stock. WB analyses of S^+^ VLPs and extracellular vesicles (EVs) produced in or spontaneously released from HEK293T cells, and SARS-CoV-2 virus stock produced from infected Vero E6 cells are shown. Antibodies are directed against the S1 or S2 domains or recognize the intact, full-length (FL) spike molecule S^FL^. The analyses confirm the presence of spike protein in various states in S^+^ VLPs and SARS-CoV-2 virion preparations but not in EVs which served as negative control. (A) and (B) S2 and S1 specific monoclonal respective polyclonal antibodies detect both spike domains in cell-free preparations of S^+^ VLPs as well as S^FL^ protein (left panels of A and B). The S2 domain specific antibody also detects trimeric S^FL^ (S^FL^_3_) and spike complexes of higher order under nonreducing (nonred) conditions. In SARS-CoV-2 virus stock (right panels of A and B) the antibodies detect S^FL^ protein and the S1 domain in panels A and B but not the S2 domain. (C) Mono- and trimeric S^FL^ protein complexes in S^+^ VLPs (left panel) and SARS-CoV-2 virus stock (right panel) detected with 43A11, a monoclonal antibody that recognizes full-length spike (S^FL^) exclusively.

Because of its central role in viral infection, the correct conformation of S is critical for the tropism and fusogenicity of both SARS-CoV-2 virions and S^+^ VLPs. We used two commercially available antibodies that recognize S1 or S2 together with 43A11, a new, in-house generated high affinity monoclonal antibody (Fig. S1, Supplementary Material), which exclusively recognizes nondissociated S (but not the single S1 or S2 domains; Fig. 2C) to visualize S1, S2, S full-length (S^FL^), and higher order S^FL^ complexes in S^+^ VLP preparations under reducing and nonreducing conditions. By western blot (WB) analysis (Fi-g. 2), we confirmed the presence of S1 and S2 domains, S^FL^, trimers of S^FL^ (S^FL^_3_ or S2_3_S1_3_), and other additional S complexes in our S^+^ VLPs preparations. Proteolytic cleavage by furin and possible subsequent dissociation of S1 during viral egress (40, 41), but also dimeric S^FL^ complexes might generate certain additional higher order complexes (42), which we also observed in Figure 2. In S^+^ VLP preparations from HEK293T cells the majority of S is efficiently cleaved, presumably by furin (Fig. 2B, left panel, reducing conditions), but S1 and S2 domains remain largely complexed in single and higher order S^FL^ conformations (Fig. 2A and C, left panels, nonreducing conditions).

Parallel to S^+^ VLPs, we analyzed a heat inactivated (56°C, 15 min) SARS-CoV-2 virus stock harvested from infected Vero E6 cells. Compared to S^+^ VLPs, the virus stock showed a similar S composition, but the S2-specific antibody recognized only S^FL^, whereas the S1 antibody detected both S^FL^ and S1. Using the 43A11 monoclonal antibody (mAb), the SARS-CoV-2 virus stock was found to contain S in its distinct trimeric state, but also monomeric S^FL^. Repeated passaging of SARS-CoV-2 on Vero E6 cells can lead to the loss of the furin cleavage site as has been previously reported (43), which might explain the equal fraction of noncleaved S (Fig. 2B, right panel) and the absence of the separate S2 domain in the virus stock in Figure 2A. We conclude that our S^+^ VLP preparations and the SARS-CoV-2 virus stock are similar according to WB analyses but differ with respect to the fraction of furin cleaved S.

**Fig. 3.**
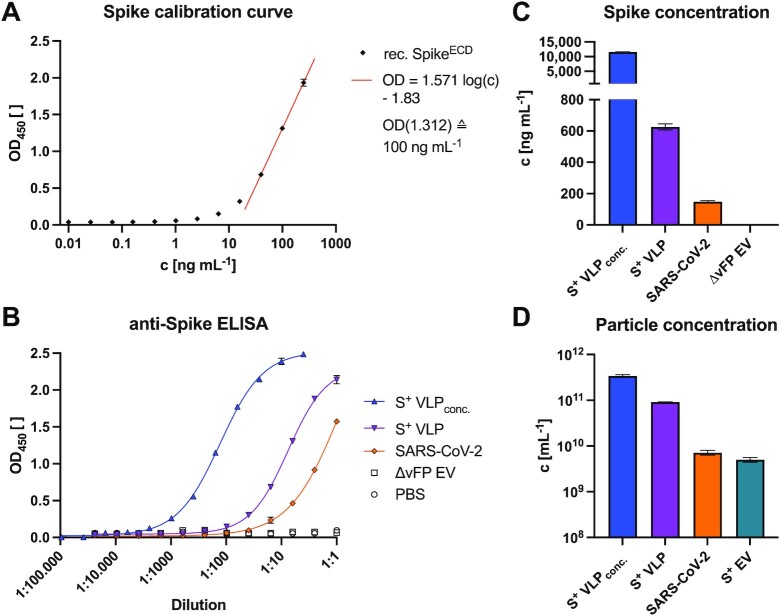
Spike-specific, quantitative sandwich ELISA, and NTA of S^+^ VLP preparations and heat-inactivated SARS-CoV-2 virus stock. To quantify spike protein in biological samples, a sandwich ELISA was established with two mAbs (43A11 and 55E10) that recognize two orthogonal, nonoverlapping epitopes in S^FL^ protein. Mean values with standard deviations are indicated. (A) Calibration of the sandwich ELISA with a commercially available recombinant (rec.) S protein standard, encompassing the extracellular domain (ECD) of spike. The calibration curve of three independent replicates allows for calculating the amount of S protein in samples within the linear range of optical density (OD) values (0.7 ≤ OD ≤ 1.7, *r*^2^ > 0.99). The detection limit of this assay was estimated to correspond to 3 ng mL^−1^ recombinant S protein. (B) and (C) Concentrated (conc.) and nonconcentrated S^+^ VLPs from supernatants of transiently transfected HEK293T cells were analyzed for their amount of S^FL^ protein. Based on the linear regression function in panel A, the concentration (c) of S was calculated (ng mL^−1^) of three technical replicates in the linear OD range and compared with inactivated SARS-CoV-2 virus stock with a known ct value (15.3) of its vRNA copies according to RT-qPCR. Controls are solvent (PBS) and EVs without a viral FP (∆vFP EVs) harvested from cell culture medium of HEK293T cells transiently transfected with expression plasmids coding for M, N, E, and CD63∼HiBiT but omitting S. (D) NTA of three independent preparations of unconcentrated and concentrated S^+^ VLPs (S, M, N, E, and CD63∼HiBiT) and S^+^ EVs (S, CD63∼HiBiT omitting M, N, and and E) from cell culture medium of transiently transfected HEK293T cells are shown. For comparison, NTA data from heat-inactivated SARS-CoV-2 virus stock from infected Vero E6 cells are provided.

We also developed a highly sensitive sandwich enzyme-linked immunosorbent assay (ELISA) to characterize S^+^ VLP and the SARS-CoV-2 virus preparations and to quantify their S content. We used the mAb 43A11 together with 55E10, a second in-house generated anti-S mAb (Fig. S1A, Supplementary Material), which recognize orthogonal, nonoverlapping epitopes. As external reference we employed a commercially available recombinant S protein to obtain a calibration curve to assess the amount of S (Fig.   3A). The assay reliably detected S concentrations as low as 3 ng mL^−1^ recombinant protein (Fig. 3A) as well as S protein in S^+^ VLP preparations (Fig. 3B), and was found to be highly specific when probed with control samples consisting of extracellular vesicles (EVs) without a viral FP (∆vFP EVs). Using this ELISA, we quantified our S^+^ VLP productions and heat inactivated SARS-CoV-2 virus stock (RT-qPCR, ct value of 15.3) and found about 626 ± 20 ng mL^−1^ and 149 ± 6 ng mL^−1^ S protein, respectively, according to the S protein standard (Fig. 3C).

### Particle analysis of SARS-CoV-2 VLPs

Next, we quantified the number of physical particles in our samples via nanoparticle tracking analysis (NTA). Intriguingly, coexpression of M, N, and E together with S increased the number of particles drastically compared to transfections of S alone (Fig. 3D), suggesting that SARS-CoV-2 S^+^ VLPs consisting of M, N, E, and S evolve via a self-assembling mechanism and egress without the need of nonstructural viral proteins, as described for SARS-CoV VLPs (44). NTA of our samples indicated that our S^+^ VLP preparations contained 9.1 × 10^10^ mL^−1^ particles, while comparable preparations of EVs obtained from HEK293T cells after transfection with the S encoding expression plasmid (S^+^ EV in Fig. 3D) only yielded 5.0 × 10^9^ mL^−1^ particles. The heat inactivated SARS-CoV-2 virus stock was determined to contain 7.2 × 10^9^ mL^−1^ particles. Total particle counts included 1.5 × 10^9^ mL^−1^ bovine EVs from fetal bovine serum (FBS) contained in cell culture medium. Bovine EVs corresponded to 2% and 30% of total particle numbers in S^+^ VLP and S^+^ EV preparations, respectively.

**Fig. 4.**
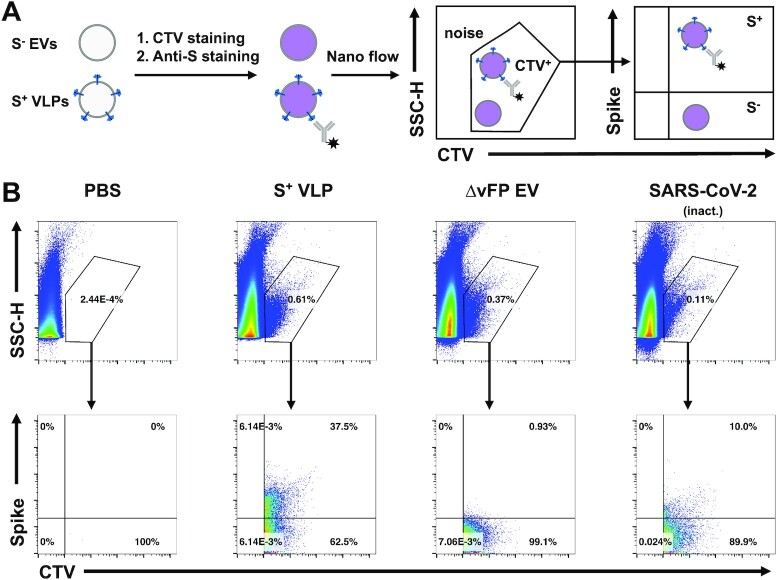
Nano flow technology of S^+^ VLPs and heat-inactivated SARS-CoV-2 virus stock. (A) HEK293T cells were transfected with S, M, N, E, and CD63∼HiBiT or with M, N, E, and CD63∼HiBiT but without S to produce S^+^ VLPs or control ∆vFP EVs, respectively. After two rounds of low-speed centrifugation, cell culture supernatants containing either S^+^ VLPs or control ∆vFP EVs were stained with the membrane permeable dye CTV, which exhibits fluorescence only upon its uptake followed by esterase activation within the lumen of intact membranous vesicles. A heat-inactivated SARS-CoV-2 virus stock was also stained with CTV for comparison. Subsequently, samples were counter-stained for the presence of surface spike protein using the monoclonal anti-S antibody 43A11. The samples were diluted and analyzed using a CytoFLEX LX flow cytometer. (B) Panels in the top row show recorded events according to their sideward scatter (SCC-H) using a violet excitation laser (*y*-axis) and CTV staining (*x*-axis). CTV^+^ events were gated as shown to identify subcellular, intact particles (S^+^ VLPs, ∆vFP EVs, and SARS-CoV-2 virus) to distinguish them from instrument noise seen in the PBS control. CTV^+^ events were analyzed for their staining with the anti-S antibody 43A11 coupled to AlexaFluor488 (bottom row of panels). 37.5% S^+^ particles were identified in the preparation of S^+^ VLPs, 10% S^+^ particles were identified in SARS-CoV-2 virus stock and fewer than 1% in control ∆vFP EVs. The low fraction of CTV-positive events in SARS-CoV-2 virus stock (0.11%) compared with preparations of S^+^ VLPs (0.61%) and ∆vFP EVs (0.37%) might be the consequence of a reduced esterase activity in virions (and EVs) after heat inactivation at 56°C for 15 min to inactivate viral infectivity.

To obtain quantitative data of our S^+^ VLPs at the level of single particles, we developed a nano flow technique to assess the fraction of S-positive particles among all particles released by HEK293T cells. Particles were purified from cell culture supernatants and incubated with the dye CellTraceViolet (CTV; Thermo Fisher Sci.), which exhibits fluorescence upon enzymatic ester hydrolysis in the lumen of intact vesicles after membrane penetration. Subsequently, particles were stained with the fluorescently labeled anti-S mAb 43A11 and analyzed by our nano flow technology using a cytometer (CytoFLEX, Beckman Coulter). To distinguish instrument noise from particles we pre-gated on CTV^+^ events and SSC-H (Fig. 4A) and measured the fraction of S-positive particles eventually. S^+^ VLPs, i.e. particles obtained after transient transfection of HEK293T cells with the plasmids encoding S, M, N, and E, constituted 37.5% of all CTV^+^ particles. The inactivated SARS-CoV-2 stock contained about 10% S-positive particles (Fig. 4B). Thermal treatment of the SARS-CoV-2 sample might have lowered the esterase activities in both virions and EVs, which might explain the generally lower fraction of CTV^+^ particles (0.11% of all events) in this SARS-CoV-2 virus stock (top panels in Fig. 4B). We, therefore, estimated the fraction of SARS-CoV-2 virions and S^+^ VLPs to be in a comparable range but S^+^ VLPs were abundant by a factor of three or more.

Our preparations of S^+^ VLPs were found to contain on average 626 ng S protein (Fig. 3C) and 9.1 × 10^10^ physical particles per mL (Fig. 3D) of which 37.5% carried spike (Fig. 4B). Assuming even distribution of S molecules per S^+^ particles and the given molecular weight of 134.4 kDa for the truncated recombinant S protein standard, we calculated a theoretical number of 82 S molecules, corresponding to 27 trimers per S^+^ VLP. This number is in the range described by others for SARS-CoV-2 (36, 37, 43).

### Virus-free neutralization test

Toward a virus-free neutralization test we analyzed the capacity of S^+^ VLPs to fuse with appropriate target cells. First, we transduced various cell lines (HEK293T, LN18, A549, Huh7, Vero, and U251MG) to constitutively express human ACE2 and validated its expression by WB. Of these cell lines, Huh7 and Vero cells had previously been used for infection with spike-pseudotyped retrovirus vectors (45). Recently, we identified the SARS-CoV-2 susceptible human cell line U251MG (46), to take up EVs equipped with vesicular stomatitis virus glycoprotein G (VSV-G) very efficiently suggesting that these cells might also be suitable to act as S^+^ VLP recipient cells (47).

Next, we engineered EVs to contain SARS-CoV-2 spike together with CD63∼BlaM (a chimer of human CD63 and β-lactamase), concentrated them via ultracentrifugation and incubated them with the panel of recipient cells as described above for 4 h. Upon uptake and fusion of these EVs with recipient cells, BlaM translocates to the cytoplasmic compartment of the recipient cells. Only BlaM^+^ cells loaded with the CCF4-AM substrate convert it to fluorescent CCF4, which can be quantified via flow cytometry on single cell level as described in the context of HIV-1 (48), and later by us (47) and others (49–53).

ACE2^+^ Vero cells took up S^+^ EVs but not control EVs lacking a viral FP (∆vFP EVs; Fig. S2A, Supplementary Material). Yet only about 21% of Vero cells turned BlaM-positive even with a high dose of S^+^ EVs, while 75% of all cells became positive with control EVs equipped with CD63∼BlaM and VSV-G as a viral FP (VSV-G^+^ EVs; Fig. S2B, Supplementary Material). In contrast, up to 97% ACE2^+^ U251MG cells became BlaM-positive with S^+^ EVs, while cells incubated with ∆vFP EVs remained BlaM negative (Fig. S2C and D, Supplementary Material). The fusion of ACE2^+^ U251MG cells with S^+^ or VSV-G^+^ EVs was equally efficient in this setting (Fig. S2C and D, Supplementary Material). To demonstrate the endosomal uptake and fusion of EVs with the recipient cells, we pretreated ACE2^+^ Vero cells with chloroquine prior to incubation with S^+^ EVs (Fig. S2E, Supplementary Material). Chloroquine deacidifies endosomes and inactivates the endosomal-pH-dependent cysteine protease CTSL, which primes S for SARS-CoV-2 entry in certain cell lines in vitro (7, 54, 55). Thus, delivery of BlaM was found to be dependent on endosomal processing of S in Vero cells.

Next, we used ACE2^+^ U251MG cells in preliminary neutralization experiments to test and quantify the reduced uptake of S^+^ EVs by neutralizing serum antibodies. Sera from SARS-CoV-2 vaccinees and COVID-19 convalescent patients displayed dose-dependent neutralization, while sera from healthy and naïve donors barely showed any effect (Fig. S2E, Supplementary Material). We concluded that all steps of the S-mediated and ACE2-dependent cellular uptake of S^+^ EVs are highly reminiscent of SARS-CoV-2 infection.

### Optimization of the VLP fusion assay

The applicability of the assay described above is limited as its readout relies on flow cytometry, requires an overnight incubation step for the intracellular accumulation of CCF4, and depends on concentrated S^+^ EVs preparations. We, therefore, developed this assay further by replacing BlaM with nano-Luciferase (nLuc). To avoid known background problems due to protein leakage of intact nLuc, we adapted its split variant consisting of an incomplete and inactive nLuc polypeptide (LgBiT) and the self-associating activator peptide of 11 amino acids (HiBiT) for our purposes (35, 56, 57).

Similar to BlaM, we fused HiBiT to the C terminus of CD63 (CD63∼HiBiT) (58) to be incorporated into S^+^ particles. As recipient cells, we engineered ACE2^+^ U251MG cells to constitutively express *N*-myristoylated LgBiT (NM∼LgBiT) as membrane-associated reporter enzyme. This system proved to be almost free of leakage as HiBiT and LgBiT are tightly associated with the cellular and EV membranes, respectively, and are not secreted in detectable amounts. In the split nLuc system, the turnover of a suitable substrate is only catalyzed upon successful intracellular reconstitution of both parts of nLuc (56). As a consequence, this assay has a very low background, which otherwise is a major problem when working with fully active enzymes as protein reporters.

**Fig. 5.**
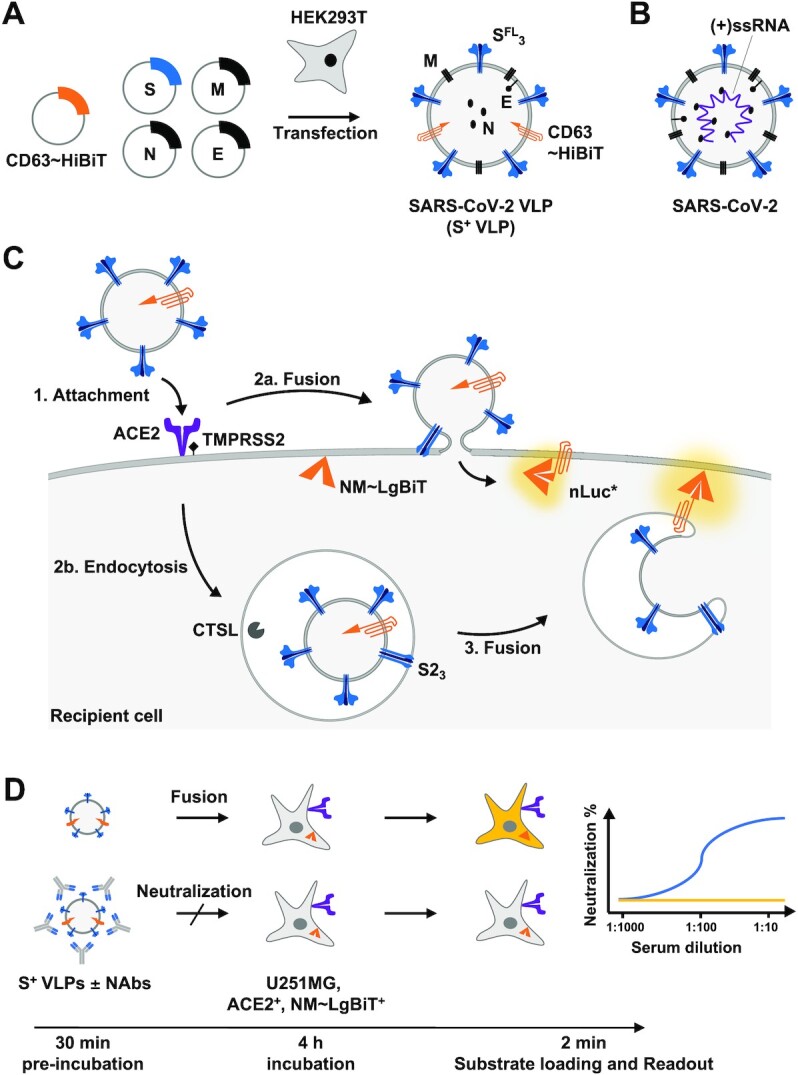
VLPNT. (A) Engineered VLPs were generated in vitro by transient cotransfection of HEK293T cells with an optimized ratio of expression plasmids encoding the four SARS-CoV-2 structural proteins S, M, N, E, and a chimeric membrane anchored activator peptide (CD63∼HiBiT). The resulting particles were termed S^+^ VLPs and obtained from conditioned cell culture medium 3 days after DNA transfection. (B) Schematic view of a SARS-CoV-2 virion with the four structural proteins S, M, N, and E and the viral genome of positive sense, single-stranded RNA [(+)ssRNA] complexed with N. (C) Basic steps of VLP entry and reconstitution of nano Luciferase (nLuc). Similar to infection with SARS-CoV-2, spike, the trimeric viral FP in the envelope of S^+^ VLPs (Fig. 1) mediates attachment (step 1) to the host cell receptor ACE2, triggering either proteolytic processing by TMPRSS2 and direct fusion at the plasma membrane (step 2a) or endocytosis (step 2b), cleavage by CTSL and subsequent fusion with the endosomal membrane (step 3). Fusion of the S^+^ VLP envelope with cellular membranes via both pathways expose the HiBiT activator peptide to make contact with *N*-myristoylated LgBiT (NM∼LgBiT), which is stably expressed in the cytoplasm of the ACE2^+^ target cell. Upon in situ reconstitution of the functional nano Luciferase (nLuc*) reporter addition of substrate will induce bioluminescence, which can be quantified in a standard luminometer in 96-well cluster plates. (D) To test body fluids for the content of neutralizing SARS-CoV-2 antibodies (NAbs), S^+^ VLPs are preincubated with serial dilutions of the samples for 30 min. Suitable medical samples are sera of COVID-19 patients, vaccinated or naïve individuals or other body fluids such as saliva or nasal excretions. SARS-CoV-2 NAbs will interfere with all steps of S^+^ VLP attachment to ACE2, receptor-mediated intake, endosomal fusion of the VLP envelope with the endosome, and escape to the cytoplasm. Target cells are U251MG cells engineered to express both ACE2 and NM∼LgBiT (LgBiT). Upon encounter with S^+^ VLP-borne CD63∼HiBiT, NM∼LgBiT is reconstituted into a fully functional nLuc reporter enzyme, which can be quantitated. Neutralizing SARS-CoV-2 antibodies reduce or even block the delivery of the CD63∼HiBiT activator entirely, which can be quantified in a standard clinical laboratory with aid of a luminometer and within 4.5 h. A freely accessible scientific animation narrates the principle of the VLPNT (https://youtu.be/6wckXobT_bM).

We also turned from S^+^ EVs to SARS-CoV-2 VLPs, i.e. S^+^ VLPs encompassing all four structural SARS-CoV-2 proteins, because coexpression of M, N, and E together with S and CD63∼HiBiT (Fig. 5A) led to higher particle numbers and enhanced fusogenicity of VLPs (Fig. 3D). We determined the optimal stoichiometry of plasmid DNAs in this practical, readout-based approach to obtain S^+^ VLPs, which resemble SARS-CoV-2 in many aspects as documented by cryo-EM, WB, ELISA, NTA, and nano flow technology as shown in Figures 1 to 4. The engineered S^+^ VLPs contact ACE2^+^ recipient cells in a receptor-dependent manner, require correct processing of S by proteases and enter cells by endocytosis followed by endosomal escape or plasma membrane fusion via the postfusion conformation of S, very reminiscent of infectious SARS-CoV-2 virions. Upon fusion the membrane anchored HiBiT is delivered into the cytoplasm of the LgBiT^+^ recipient cell, where the functional enzyme reconstitutes in situ to support substrate turnover and emission of light as shown schematically in Figure 5C. A scientific animation demonstrates this simple principle ( https://youtu.be/6wckXobT_bM).

**Fig. 6.**
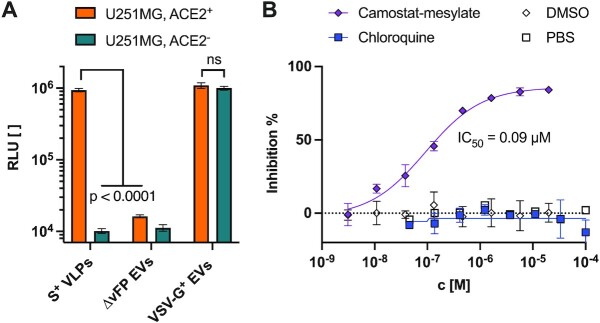
Specificity and tropism of S^+^ VLPs. (A) Two cell line derivatives of U251MG cells, which express NM∼LgBiT with or without human ACE2 receptor (ACE2^+^ or ACE2^−^) were incubated with S^+^ VLPs carrying CD63∼HiBiT, with EVs without a viral FP (ΔvFP EVs) obtained from supernatants of HEK293T cells after transient transfection of expression plasmids encoding M, N, E, and CD63∼HiBiT (but not S) or with EVs from HEK293T cells after transient transfection of expression plasmids encoding CD63∼HiBiT and protein G of the vesicular stomatitis virus (VSV-G^+^ EVs). The specificity of spike-mediated, ACE2-dependent fusion of all three particle classes was validated measuring luciferase activities upon reconstitution of the split nLuc in the indicated cell types. Data are based on at least four independent experiments. *P*-values of independent t tests are indicated (ns; not significant). (B) Inhibitor studies with chloroquine, an inhibitor of endosomal acidification and of CTSL and camostat-mesylate, a TMPRSS2 inhibitor, are shown using S^+^ VLPs and ACE2^+^ U251MG cells. DMSO and PBS served as negative controls for camostat-mesylate and chloroquine, respectively. Mean values of three biological replicates are displayed with error bars indicating standard deviations.

Analogous to the EV system with BlaM as reporter protein, we assessed whether S^+^ VLPs fuse exclusively with susceptible ACE2^+^ cells via the viral entry factor S. We generated S^+^ VLPs (S, M, N, E, and CD63∼HiBiT)^+^, VSV-G^+^ EVs (VSV-G and CD63∼HiBiT)^+^, and ∆vFP EVs (M, N, E, and CD63∼HiBiT)^+^, incubated them with ACE2^+^ or ACE2^−^ NM∼LgBiT^+^ U251MG cells for 4 h and quantified their fusion with the different target cells. As expected, S^+^ VLPs fused exclusively with ACE2^+^ cells but not with ACE2^−^ cells (Fig. 6A). Furthermore, fusion relied strictly on the presence of a viral FP such as S because ∆vFP EVs were barely taken up. In contrast, VSV-G^+^ EVs fused with ACE2^+^ and ACE2^−^ cells at similar levels due to VSV-G's broad tropism (Fig. 6A). To elucidate the entry pathway of S^+^ VLPs (S: D614G), we pretreated U251MG cells either with chloroquine or with the serin protease inhibitor camostat-mesylate (Fig. 6B), which inhibits TMPRSS2 (59). In contrast to our results with Vero cells (Fig. S2E, Supplementary Material), chloroquine did not reduce S^+^ VLP entry into U251MG cells, but camostat-mesylate efficiently inhibited S^+^ VLPs entry with an IC_50_ of 0.09 µM. Camostat-mesylate is known to block TMPRSS2 dependent entry of SARS-CoV-2 in certain cell-lines in vitro (7, 55). We conclude that this system meets all requirements of a S^+^ VLP-based neutralization test.

### VLPNT

S-specific NAbs can prevent entry of SARS-CoV-2 virions into host cells, and thus protect from viral infection via diverse modes of actions. Most antibodies block the attachment of S to ACE2 receptors by binding to the RBM of S1 (60, 61) or stall S in its closed, i.e. RBD “down” prefusion conformation (62). Yet, certain S-specific NAbs also neutralize without disrupting the ACE2 interaction (63). Possible other mechanisms include the inhibition of proteolytic processing of S by TMPRSS2 or CTSL or the interference with the heptad repeats or glycosylated surfaces in S2, which are required to promote the fusion of the viral envelope with the endosomal membrane as for SARS-CoV or MERS-CoV (64, 65). NAbs with their multiple mechanisms to disrupt S functions also reduce, interfere with, or even block S^+^ VLPs, and thus the delivery of CD63∼HiBiT to susceptible target cells. As a result, reduction of luminescence from reconstituted nLuc might likely correlate with SARS-CoV-2 neutralization (Fig. 5D).

**Fig. 7.**
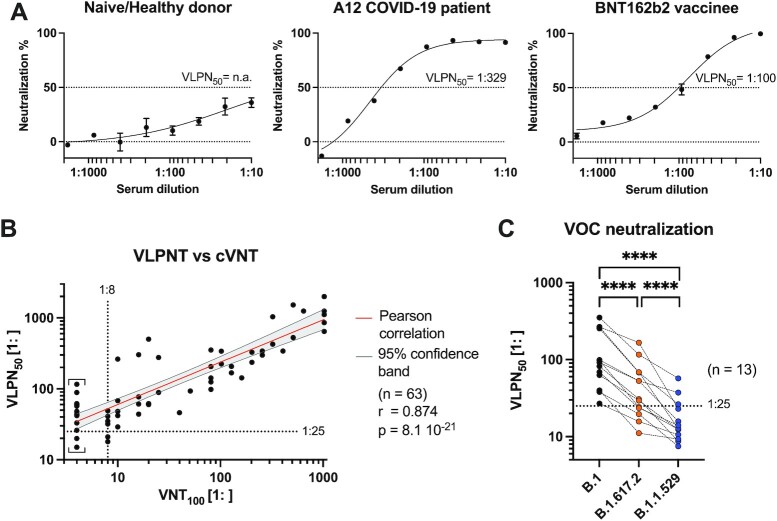
Correlation of VLPNT and cVNT data and VOC cross-neutralization using sera from COVID-19 patients and vaccinees. (A) VLPNT neutralization data with dilutions of sera obtained from three individuals (a naïve healthy donor, the COVID-19 patient A12, and a BNT162b2 vaccinee) are shown. The graphs are examples and include mean neutralization results from three independent biological replicates. Serum dilution which resulted in half maximal signal reduction, equivalent to 50% neutralization, was termed VLPN_50_ titer. (B) Correlation of VLPN_50_ titers from the VLPNT vs. VNT_100_ titers obtained in a cVNT using infectious SARS-CoV-2. Pearson correlation data (sample size *n*, coefficient *r*, and *P*-value) of 63 sera from confirmed COVID-19 patients are shown and the linear relationship is indicated. Results below the dotted horizontal line denote sera, which scored negative in the VLPNT. Results left of the dotted vertical line denote sera which scored below the LOD (1:8) in the cVNT; these VNT_100_ values were defined as 1:4 and indicated by square brackets. (C) VLPNT with two SARS-CoV-2 VOCs compared with the B.1 strain. S^+^ VLPs were harvested from supernatants of HEK293T cells transiently transfected with expression plasmids encoding either B.1 (S: Wuhan-2019, D614G), B.1.617.2 (Delta-VOC), or B.1.1.529 (BA.1, Omicron-VOC) S protein together with M, N, E, and CD63∼HiBiT. NAbs in sera of 13 COVID-19 vaccinees (after prime-boost vaccination) were cross-neutralizing, but less potent in neutralizing B.1.617.2 compared with B.1. The majority of serum samples however, failed to neutralize the B.1.1.529 variant effectively. Data derived from 13 samples were analyzed using a matched one-way ANOVA, with Tukey's multi comparison test and a single pooled variance. Results are indicated: ****P ≤ 0.0001.

Toward a VLP-based virus neutralization test (VLPNT), we incubated a defined amount of S^+^ VLPs with serial dilutions (starting from 1:10 to 1:1,800) of sera from acute or convalescent COVID-19 patients, COVID-19 vaccinees or healthy, naïve donors, and quantified the resulting dose-dependent neutralization as shown in Figure 7A. Mean luminescence level of S^+^ VLPs, only, was set to 0% neutralization; while background luminescence obtained with ∆vFP EVs was set to 100% neutralization. The half maximal neutralization titer was determined by extrapolating sigmoidal curve values that correspond to 50% signal reduction after background correction. This value was termed VLPN_50_. It is considered equivalent to VNT_50_ and PRNT_50_ of cVNTs and PRNTs, respectively.

Sera of naïve donors resulted in little to no signal reduction, i.e. weak neutralization even at high serum concentrations. Of 12 sera from healthy, naïve donors obtained from mid 2019 and earlier, most did not reach 50% neutralization and few showed VLPN_50_ titers of 1:16 or lower (Fig. S3A, Supplementary Material). Sera of 13 COVID-19 vaccinees after prime-boost immunization from 2021 showed high neutralization potential consistently up to 100% inhibition of S^+^ VLPs fusion and VLPN_50_ titers of 1:27 to 1:352 (Fig. S3A, Supplementary Material). Data on individual titers and types of vaccines which the donors received can be found in Figure S3D and Table S1 (Supplementary Material). Based on the maximal titers of naïve sera in our VLPNT, we added an additional margin and defined a preliminary minimal cutoff of 1: ≥ 25 to classify samples with neutralizing activities.

As there is considerable demand for global harmonization and standardization of NAb titers obtained from different laboratories using different versions of SARS-CoV-2 VNTs (66), the World Health Organization (WHO) supplies laboratories with a standard plasma pooled from 11 British patients, who recovered from COVID-19 (Mattiuzzo et al. 2020, WHO/BS/2020.2403). We applied this standard (NIBSC 20/136) with a defined neutralization activity of 1,000 international units per mL (IU mL^−1^). VLPN_50_ values of multiple individual experiments with the WHO standard were obtained (Table S1, Supplementary Material) and used to convert our serum VLPN_50_ titers to harmonized titers expressed as IU mL^−1^ as shown in Figure S3A (Supplementary Material), right panel. In six independent VLPNT experiments (including separate S^+^ VLP batches) with the WHO standard, we calculated a coefficient of variation (CV) for log_10_ transformed titers of CV = 0.04 and CV = 0.23 for untransformed titers, for the within-laboratory repeatability and the resulting imprecision of quantification, respectively. Clearly, additional experiments and standardizations have to be conducted to generate data for an assessment according to the Clinical Laboratory Standards Institute (CLSI) guidelines (67).

We also used our VLPNT to test two potent neutralizing recombinant mAbs, REGN10987, imdevimab and REGN10933, casirivimab, which have been approved under emergency use authorization for the treatment of COVID-19 by the Food and Drug Administration (FDA) (26, 68, 69). Their VLPN_50_ were found to be 1.8 nM and 7.3 nM, respectively, while a human IgG isotype control mAb did not show any neutralization (Fig. S4, Supplementary Material).

Based on these results we concluded that the VLPNT qualifies for the measurement of neutralizing activities in clinical samples.

### VLPNT and cVNT compared

For the validation of our VLPNT we compared the VLPN_50_ titers of a defined set of sera with results from a cVNT using infectious SARS-CoV-2, the “gold standard” in the field (34). Toward this end, we tested 63 well-documented serum samples from 40 patients with confirmed SARS-CoV-2 infection under double-blinded, randomized conditions in our VLPNT. Relevant details about their clinical status and neutralizing serum titers data are presented in Table S1 (Supplementary Material).

When analyzed in our test, serum VLPN_50_ titers of the 63 COVID-19 sera varied from 1:18 to 1: > 2,000 and, therefore, scattered wider than serum titers from vaccinees, even though the medians were similar (Fig. S3A, Supplementary Material, left panel). Data on the individual titers and the patients’ clinical symptoms can be found in Figure S3C (Supplementary Material). Due to the previously defined cutoff of 1: ≥ 25 for the VLPN_50_, 58 out of 63 COVID-19 samples were classified as neutralizing.

The 63 COVID-19 samples were also tested in a cVNT with replication competent SARS-CoV-2 virus, as described previously (70). The titers were determined based on 100% reduction of CPE (VNT_100_) and their values ranged from 1:8 to 1: > 1,024 (Fig. 7B). In the cVNT, 48 out of 63 samples were found to have neutralizing activity with VNT_100_ 1: ≥ 8, while the 15 remaining sera performed below the limit of detection (LOD) and were set to 1:4 per definition.

We compared the VLPNT with the cVNT by correlating the VLPN_50_ titers of all 63 COVID-19 samples with the respective VNT_100_ titers (Fig. 7B). The Pearson coefficient of log_10_ transformed data (*n* = 63) revealed a highly significant positive correlation (*r* = 0.874 and *P* = 8.1 × 10^−21^). These results indicate that the VLPNT is not only a qualitative test for the in vitro diagnostics of NAbs but also yields reliable titers of NAbs faithfully reflecting titers obtained in a cVNT with infectious SARS-CoV-2.

A comparison of the 63 COVID-19 samples in both tests found 46 sera to be concordant positive (CP), three were concordant negative (CN), whereas 14 sera were found to be discrepant (D; Fig. S3B, Supplementary Material, left panel). This discrepancy likely resulted from experimental differences between the two tests (31). The multicycle cVNT relies on 100% reduction of CPE (VNT_100_), while the VLPNT is single-cycle and scores at 50% reduction of S^+^ VLP fusion (VLPN_50_) suggesting that the latter test is more sensitive and identifies serum samples with weakly neutralizing activities. Given this uncertainty, we based the calculation of sensitivity and specificity of our test on clearly attributable positive (neutralizing) and negative (non-neutralizing) specimens, using the 48 COVID-19 samples which scored above LOD in the cVNT along with 12 sera from healthy, naïve donors from mid 2019 and earlier (Fig. S3B, Supplementary Material, right panel). Of the 48 positive samples, the VLPNT classified 46 samples true positive (TP), while all 12 negative sera were considered true negative (TN). Therefore, the sensitivity of our VLPNT was calculated to be 96%, while its specificity was 100%. Based on the analyzed data set (*n* = 60), the positive predictive value (PPV) was 100%, suggesting that our SARS-CoV-2 VLPNT has the potential to become a reliable diagnostic tool.

### Test adaptation to variants of concern

Finally, we adapted the VLPNT to test and compare three variants of concern (VOCs) of SARS-CoV-2 employing spike proteins from B.1 (Wuhan-2019 and D614G), B.1.617.2 (Delta), and B.1.1.529 (BA.1, Omicron) to generate S^+^ VLPs. Their comparison necessitates an “inter-variant” normalization of S^+^ VLP stocks. Toward this aim, we employed the pan-variant neutralizing monoclonal antibody 35B12 (Fig. S3E, Supplementary Material). The resulting VLPN_50_ of 1.05 to 0.85 nM for all three variants allowed to calculate a CV = 0.11 inbetween variants.

Interestingly, the REGN10987 and REGN10933 antibodies retained their capacity to neutralize B.1.617.2, but failed to neutralize B.1.1.529 (Fig. S4, Supplementary Material). For the WHO standard (NIBSC 20/136), a similar but progressive reduction of neutralization titers could be observed (Fig. S4, Supplementary Material).

We then retested all 13 sera of SARS-CoV-2 vaccinees (Fig. S3D, Supplementary Material) in the VLPNT with S^+^ (B.1.617.2) and S^+^ (B.1.1.529) VLPs, determined their VLPN_50_ titers and compared them to titers obtained with the B.1 variant (Fig. 7C). Pearson coefficient of log_10_ transformed data (*n* = 13) was calculated and showed strong positive correlation for B.1 vs. B.1.617.2 (*r* = 0.911; *P* = 1.5 × 10^−5^) and a reduced correlation for B.1 vs. B.1.1.529 (*r* = 0.700; *P* = 0.0077). Apparently, NAbs induced by different SARS-CoV-2 vaccines based on Wuhan-2019 spike cross-neutralized VOC B.1.617.2, but failed to cross-neutralize VOC B.1.1.529 efficiently. Further, neutralization titers significantly differed inbetween variants as shown by a matched one-way ANOVA of log_10_ transformed data (*P* < 0.0001, *n* = 13; Fig. 7C). While the mean titer for B.1 S^+^ VLPs was found to be 1:135, the mean titers for B.1.617.2 (1:52) or B.1.1.529 (1:19) were significantly reduced by factor 0.39 or 0.14, respectively (Fig. S3F, Supplementary Material). Notably, of 13 prime-boost vaccinated individuals with B.1 titers above cutoff level (1: ≥ 25), only three showed some neutralizing activity against Omicron BA.1 indicative of immune escape and reflecting its independent serotype. A reduced capacity of convalescent or vaccinee sera to cross-neutralize B.1.617.2 has been demonstrated previously in a pVNT (71) and also in a cVNT (72). For B.1.1.529, substantially reduced titers in vaccinated individuals have also been reported using cVNTs (24, 73). Thus, our findings appear to be consistent with published work. The successful adaptation of the VLPNT to VOCs indicates that our test is flexible and versatile to address and answer questions on cross-NAbs between existing and future SARS-CoV-2 VOC, likely including the currently spreading Omicron BA.2 sublineage.

## Discussion

Serum antibody levels are a predictive and easily accessible medical parameter of individual immune protection from viral infections and related diseases. For SARS-CoV-2, especially NAb titers were shown to correlate with clinical protection from COVID-19 (27). In clinical virology, the detection and quantitation of NAbs in biological samples is commonly performed with conventional VNTs (cVNTs), which mostly rely on in vitro CPEs and the formation of plaques in infected cell monolayers. NAbs interfere with cellular infection but the formation of plaques depend on replication competent virus. As a consequence, these tests require appropriate containment and regulatory measures. cVNTs with SARS-CoV-2 for instance require a BSL-3 containment. Moreover, the tests take several days and they are difficult to normalize and to standardize between different laboratories. Plaque formation requires visual microscopic inspection by trained personnel and PRNTs are, thus labor intensive and cumbersome. To overcome this problem, the focus reduction neutralization test (FRNT), also known as microneutralization assay (MNA) relies on the identification of infected cells or foci by immunostaining, but the test requires numerous steps to completion and takes several days (32, 74, 75). Therefore, surrogate methods have been invented and used, which are commonly based on pseudotyped retro- or lentiviruses so-called pVNTs. The tests, which are manageable in BSL-2 laboratories rely on de novo expression of a reporter enzyme or fluorescent protein encoded by the viral vector, but they still require up to 3 days for final assessment.

The artificial, replication deficient retro- or lentiviral vectors can be problematic because viral glycoproteins, which mediate attachment, uptake, and fusion with cellular membranes might perform differently in the context of viral vectors compared to their authentic viral host (31, 76). S-pseudotyped retro- or lentiviral vectors mostly bud from the plasma membrane where S is incorporated into the envelope of the vector with uncertain stoichiometry (76, 77). Very much in contrast, the envelopes of coronaviruses carry a discrete number of S trimers per particle, which assemble in the ER–Golgi intermediate compartment together with M, E, and N in a given, optimal stoichiometry (78).

We performed such pVNT for SARS-CoV-2, produced S-pseudotyped, replication incompetent retroviruses, preincubated them with different human sera, infected hACE2^+^ Vero cells, cultivated them for 48 h to allow expression of the GFP reporter gene and analyzed them by flow cytometry and fluorescence microscopy (Fig. S5, Supplementary Material). On average about 80% of cells were infected, while preincubation of the test vectors with sera from COVID-19 convalescent patients and COVID-19 vaccinated individuals reduced the fraction of infected cells in a dose dependent manner. Conversely, sera from healthy naïve individuals did not show an effect. pVNTs allow to quantitate NAbs with SARS-CoV-2 specificity, but they are biologically distant from infectious SARS-CoV-2 stock, they take several days and they depend on qualified S-pseudotyped virus stocks with reproducible vector content and infectivity.

Miyakawa et al. (35) considered the unfavorable time-to-readout problem of pVNTs and replaced the HIV-borne reporter gene with a reporter protein consisting of the HIV capsid protein with a carboxy-terminal HiBiT domain following the principle proposed by Cavrois et al. in 2002 (48). Similar to our approach (Fig. 5) recipient Vero cells expressed LgBiT constitutively to monitor the fusion of the S-pseudotyped HIV particles, which shortens the time-to-readout to 3 to 4 h (35). This approach, however, does not solve or address the partly moderate correlations of pVNTs and “live-virus” cVNTs observed in clinical samples with *r* values ranging from 0.31 to 0.89 for SARS-CoV and SARS-CoV-2 (28, 32, 34, 79, 80). These observations probably reflect differences in viral morphogenesis, egress, composition of S-pseudotyped retro- and lentiviral vector particles, and their mode of uptake by recipient cells compared to SARS-CoV-2 virions.

For SARS-CoV (81), and later SARS-CoV-2, VLPs were shown to mediate transport and delivery of reporter transcripts that encompass cis-acting packaging signal sequences mimicking the generation of authentic SARS-CoV-2 and their infectivity (76). This is a major achievement, but the readout still depends on de novo expression of the reporter transcript encoding luciferase in this report.

Our test overcomes the current limitations of quantitating SARS-CoV-2 NAbs as it is based on harmless, noninfectious VLPs engineered to be authentic morphological and functional mimics of SARS-CoV-2 virions. First, we optimized conditions for in vitro generation of such S^+^ VLPs and characterized them together with a sample of heat-inactivated SARS-CoV-2 stock using standard approaches together with a novel nano flow technology. By cryo-EM, we confirmed the presence of intact, native S^+^ VLPs with the characteristics of coronaviruses in our preparations. Furthermore, the S^+^ VLPs contain S^FL^ trimers, a molecular concentration of S very similar to authentic SARS-CoV-2 virions and particle number and ratio of S^+^ particles comparable to virus stocks. We calculated the number of S trimers per particle to be 27 and, therefore, well within the range of published figures which vary from 24 to 40 (36, 37, 43).

Second, we engineered S^+^ VLPs to carry a chimeric, membrane anchored, and luminally oriented enzyme (BlaM) or an activator peptide (HiBiT) and generated hACE2^+^ Vero cells and a hACE2^+^ U251MG cell line, which carries an inactive, membrane-associated split reporter nLuc enzyme (LgBiT). In quantitative fusion experiments with S^+^ VLPs we showed that only ACE2^+^ cells take up S^+^ VLPs, while ACE2^−^ cells were not susceptible as expected. Uptake strictly depended on spike, the viral entry factor, indicating that our S^+^ VLPs do not only structurally and molecularly resemble SARS-CoV-2 virions but also share their specific fusogenic characteristic and tropism. A limitation of Vero cells as recipients is that mostly CTSL but not TMPRSS2 mediates spike processing unlike in vivo infection of human lung cells with SARS-CoV-2. Efficient entry into U251MG cells, however, depends on TMPRSS2 (Fig. 6B).

With the thoroughly characterized S^+^ VLPs and our reporter system, we went on and demonstrated the proof-of-principle of a rapid and safe VLPNT (Fig. S6, Supplementary Material). We incubated S^+^ VLPs with 63 sera from COVID-19 and convalescent patients, 13 COVID-19 vaccinees and three neutralizing recombinant mAbs (REGN10987, imdevimab; REGN10933, casirivimab; and 35B12) and quantitated the resulting reduction of VLP-cell fusion. We controlled our test with 12 sera from healthy and naïve donors and an IgG isotype mAb, for which we did not find significant neutralization capacities. We quantitatively evaluated all samples to determine their individual titers. Based on the tested cohort of donors we defined a titer of 1: ≥ 25, to classify a sample to contain SARS-CoV-2 specific NAbs. Below this level matrix effects from healthy, naïve sera came into play, which is why even weaker neutralizations cannot be distinguished from artefacts. For the 63 COVID-19 samples we correlated the titers with the double-blinded results from a cVNT with SARS-CoV-2, resulting in convincing quantitative concordance. Thereby, we verified our VLPNT vs. a cVNT and found good preliminary sensitivity and specificity of all test parameters.

Determining NAb levels is of epidemiological and clinical relevancy, as a reduction below a certain threshold can impact protection from infection and increase the personal risk to develop COVID-19. Khoury et al. found that 20% of the mean NAb level detected in convalescent sera provided 50% protection from symptomatic disease (27). Based on these data, a minimal NAb titer could be specified, below which booster shots might become advisable.

Since the start of the pandemic in December 2019, the SARS-CoV-2 genome has undergone several mutations especially in the S protein. The original Wuhan-Hu-1 isolate (1) rapidly gained the D614G spike mutation (11) and evolved further to the Delta and then the Omicron VOC, which is the predominant variant in USA and Europe as of March 2022 (data of cdc.gov and ecdc.eu). In our VLPNT, emerging VOCs such as the B.1.1.529 Omicron VOC can be tested as to whether they present as immune escape mutants and resist neutralization when using sera from vaccinees or convalescent patients vaccinated, respectively infected with previous SARS-CoV-2 strains. This is because S^+^ VLPs that contain newly identified spike variants can be produced and validated rapidly. Along this line, we reanalyzed sera from 13 vaccinees using S^+^ VLPs with B.1.617.2 or B.1.1.529 spike and found cross-neutralization but also a significant reduction of neutralization titers concordant with published findings (71–73, 82). In principle, our VLPNT should also be capable of identifying antibody dependent enhancement effects (ADE), which are known for other viruses and has also been reported in the context of SARS-CoV-2 infection (83).

In summary, we present a rapid and safe virus-free yet authentic test to quantitate SARS-CoV-2 NAbs. The structurally and biochemically well characterized S^+^ VLPs replicate the initial steps of SARS-CoV-2 infection and allow their quantitative analysis. The VLPNT correlates very well when put to the test with a set of COVID-19 patient's sera analyzed in a benchmark cVNT with fully infectious, calibrated SARS-CoV-2 stock. The technology likely fulfills important automatization and upscaling criteria to be suitable for high-throughput screening approaches searching for potential neutralizing mAbs or antiviral-entry drugs in laboratories with low biosafety levels. In principle, this assay is adaptable to other enveloped viruses such as Dengue virus, West Nile Virus, Respiratory Syncytial Virus, Epstein-Barr virus, and cytomegalovirus and could also play an important role in disease preparedness. Additionally, the VLPNT is a platform technology that could simplify the testing of samples in clinical studies during vaccine development and allow for the immune status surveillance among, e.g. healthcare workers or people at risk.

## Material and Methods

### Patient samples and specimens

Serum samples and clinical data from COVID-19 patients, healthy individuals, and vaccinees were analyzed as described in SI Materials and Methods. COVID-19 patients and vaccinees are part of the COVID-19 Registry of the LMU Klinikum (CORKUM, WHO trial id DRKS00021225) and informed consent was obtained from all the vaccinees. The study was approved by the ethics committee of the Faculty of Medicine of the LMU. Residual, pre-pandemic serum samples were obtained prior to mid-2019 from 12 individuals who had consented for a virological diagnostic test. These samples were anonymized prior to use in the current study.

### SARS-CoV-2 VLPs and other engineered EVs

HEK293T cells were transiently transfected with optimized ratios of expression plasmids encoding codon-optimized VSV-G, SARS-CoV-2 M, N, E, and S together with CD63∼HiBiT or CD63∼BlaM. A total of 3 days after DNA transfection S^+^ VLPs, EVs and control samples were harvested from cell culture medium, purified, and concentrated as described in SI Materials and Methods.

### Biochemical and biophysical analyses of S^+^ VLPs and EVs

S^+^ VLPs and EVs were analyzed by standard techniques (ELISA and WB analysis) using commercial and proprietary poly- and mAbs. The physical concentration of particles were determined by NTA. Details can be found in SI Materials and Methods.

### Nano flow technology

Single VLPs and EVs were analyzed after intraluminal staining with cell trace violet (Thermo Scientific) followed by surface staining with a spike specific antibody using a CytoFLEX LX cytometer (Beckman Coulter Life Science). Details are described in SI Materials and Methods.

### Cryo-EM

Aliquots from crude or purified S^+^ VLPs were deposited on EM grids coated with a perforated carbon film and plunged into liquid ethane. Grids were analyzed with a Tecnai F20 microscope (FEI, USA) at 200 kV. Details are described in SI Materials and Methods.

### VLPNT with nanoLuciferase readout

Serial dilutions of serum samples were prepared, mixed with S^+^ VLPs, preincubated for 30 min, and incubated with U251MG (hACE2^+^ and NM∼LgBiT^+^) recipient cells at 37°C for 4 h. Upon replacement of the supernatant with substrate, bioluminescence was immediately quantified in a CLARIOstar Plus reader (BMG Labtech). SI Materials and Methods and Figure S6 (Supplementary Material) provide a detailed protocol.

### SARS-CoV-2 neutralization test (VNT_100_)

Human sera were serially diluted, 100 plaque-forming units of SARS-CoV-2 stock (German isolate BavPat1/2020) were added, incubated at 37°C for 1 h and 2 × 10^4^ Vero C1008 cells were added. After 4 days, CPE was evaluated by light microscopy. Details are available in SI Materials and Methods.

## Supplementary Material

pgac045_Supplemental_File

## Data Availability

All data are contained within our manuscript or within Supplementary Information. On two occasions in the main text we inform the reader about this fact.
