# Correction to: Quantitation of SARS-CoV-2 neutralizing antibodies with a virus-free, authentic test

**DOI:** 10.1093/pnasnexus/pgad477

**Published:** 2024-01-30

This is a correction to: Johannes Roessler, Dagmar Pich, Manuel Albanese, Paul R Wratil, Verena Krähling, Johannes C Hellmuth, Clemens Scherer, Michael von Bergwelt-Baildon, Stephan Becker, Oliver T Keppler, Alain Brisson, Reinhard Zeidler, Wolfgang Hammerschmidt, Quantitation of SARS-CoV-2 neutralizing antibodies with a virus-free, authentic test, *PNAS Nexus*, Volume 1, Issue 2, May 2022, pgac045, https://doi.org/10.1093/pnasnexus/pgac045

During a retroactive audit conducted by *PNAS Nexus*, it was discovered that this paper had an incomplete statement acknowledging compliance with the *PNAS Nexus* Human and Animal Participants and Clinical Trials policy:

Serum samples and clinical data from COVID-19 patients, healthy individuals, and vaccinees were analyzed as described in SI Materials and Methods. COVID-19 patients and vaccinees are part of the COVID-19 Registry of the LMU Klinikum (CORKUM, WHO trial id DRKS00021225) and informed consent was obtained from all the vaccinees. The study was approved by the ethics committee of the Faculty of Medicine of the LMU. Residual, pre-pandemic serum samples were obtained prior to mid-2019 from 12 individuals who had consented for a virological diagnostic test. These samples were anonymized prior to use in the current study.

The original article has been corrected and additional clarifying information regarding study participants has been added to the Material and Methods section in the Supplementary Material.

