# Supplementary material for: Quantitation of SARS-CoV-2 neutralizing antibodies with a virus-free, authentic test
Source: PNAS Nexus. 2022 Apr 14;1(2):pgac045. doi: 10.1093/pnasnexus/pgac045 (PMC9645495; doi:10.1093/pnasnexus/pgac045)
Supplement: pgac045_Supplemental_File [file pgac045_supplemental_file.pdf]

Quantitation of SARS-CoV-2 neutralizing antibodies with a virus-free, authentic test

Supporting Information

Johannes Roessler[1,2,3], Dagmar Pich[2,3], Manuel Albanese[3,4], Paul R. Wratil[3,4], Verena Krähling[5,6], Johannes Christian Hellmuth[7,8], Clemens Scherer[8,9], Michael von Bergwelt-Baildon[7,8,10], Stephan Becker[5,6], Oliver T. Keppler[3,4,8], Alain Brisson[11], Reinhard Zeidler[1,2,3] and Wolfgang Hammerschmidt[2,3*]

[1]Department of Otorhinolaryngology, University Hospital, Ludwig-Maximilians University Munich, Munich, Germany

[2]Research Unit Gene Vectors, Helmholtz Zentrum München, German Research Center for Environmental Health, Munich, Germany

[3]German Centre for Infection Research (DZIF), Partner site Munich, Germany

[4]Max von Pettenkofer Institute and Gene Center, Virology, National Reference Center for Retroviruses, Faculty of Medicine, Ludwig-Maximilians University Munich, Munich, Germany

[5]Institute of Virology, Faculty of Medicine, Philipps University Marburg, Germany

[6]German Centre for Infection Research (DZIF), Partner site Giessen-Marburg-Langen, Marburg, Germany

[7]Department of Medicine III, University Hospital, Ludwig-Maximilians University Munich, Germany

[8]COVID-19 Registry of the LMU Munich (CORKUM), University Hospital, Ludwig-Maximilians University Munich, Germany

[9]Department of Medicine I, University Hospital, Ludwig-Maximilians University Munich, Germany

[10]German Cancer Consortium (DKTK), Munich, Germany

[11]UMR-CBMN CNRS-University of Bordeaux-INP, Pessac, France

*Corresponding author
Wolfgang Hammerschmidt
Feodor-Lynen-Str. 21
D-81377 München
Germany
Phone: +49-89-3187-1506
Fax: +49-89-3187-4225
E-mail: hammerschmidt@helmholtz-muenchen.de

Keywords: SARS-CoV-2, coronavirus, virus-like particle, virus neutralization test, antibody, diagnostic test, reporter protein, variant of concern, B.1.617.2, Delta, B.1.1.529, BA.1, Omicron, correlate of protection, cryo-electron microscopy, extracellular vesicles, virosome

Material and Methods

Cell lines and cell culture

HEK293T (CID3915); HEK293, S^+ (CID4618); U251MG, $hACE2^+$ (CID4663); U251MG, $hACE2^{+,}$ $NM{\sim}LgBiT^+$ (CID4697) and Vero, $hACE2^+$ (CID4608) cells were maintained in DMEM medium (Gibco, Thermo Fisher Scientific), supplemented with 8% FBS (AC-SM-0143, Anprotec) and Pen/Strep (Gibco, Thermo Fisher Scientific). Cells were cultivated at 37°C in a water-saturated atmosphere with 5% CO_2. Cell viability was monitored by trypan blue exclusion and cultures with more than 95% viable cells were used in all experiments. Cell lines constitutively expressing S, hACE2 or NM~LgBiT were generated by retroviral transduction or transient transfection of parental cells with expression plasmids followed by selection with the respective antibiotic and flow cytometric cell sorting.

SARS-CoV-2 VLPs and other engineered EVs

To generate S^+ VLPs containing appropriate levels of SARS-CoV-2 spike (D614G or B.1.617.2) and control-EVs, HEK293T cells were transfected using TransIT-293 (MIR2700, Mirus) according to the manufacturers protocol with carefully adjusted ratios of codon-optimized expression vectors coding for S, VSV-G, or mock together with CD63~HiBiT or CD63~BlaM and, optionally, M, N and E. The expression plasmids are termed p7413.1 (S:D614G) or p7487.IA1 (S:B.1.617.2), p5451 (VSV-G), p5025 (mock), p7447.SA11 (CD63~HiBiT), p7200.2 (CD63~BlaM), p7395.LA3 (M), p7396.NA9 (E), p7391.MA5 (N), respectively. Media exchange was performed 6 h after the addition of the transfection mix to reduce a potential carry-over of plasmid DNA. After 72 hours, S^+ VLPs, $VSV\text{-}G^+$ EVs or control EVs were harvested from cell culture supernatants (DMEM, 8% FBS, supplemented with Pen/Strep, Gibco, Thermo Fisher Scientific) after low-speed centrifugation at 7°C for 10 min at 300×g and 20 min at 4200×g and generally used without further processing. If necessary and as indicated, S^+ VLP and EV supernatants were concentrated by ultrafiltration with 100 kDa cutoff or sedimented by ultra-centrifugation at 100,000×g, 4°C, 2 h followed by floatation in a discontinuous iodixanol (Optiprep, Sigma-Aldrich) density gradient (bottom to top: 1.75 mL sample with 34% final Optiprep concentration; 1.65 mL 30% Optiprep in PBS; 0.6 mL PBS) and ultra-centrifugation at 160,000×g at 4°C for 4 h. Collected fractions were washed with PBS by ultra-centrifugation at 100,000×g, 4°C, 2 h, and resuspended. Particle preparations were flash frozen in liquid nitrogen for storage at -80°C and subsequently characterized by WB, NTA, nano flow technology, ELISA and cryo-EM.

Western blot

Concentrated S^+ VLPs or EVs were lysed in non-reducing 5× Laemmli buffer and separated by 10% SDS-PAGE. Proteins were transferred to nitrocellulose membranes (GE Healthcare Life Science) by semi-dry blotting (Bio-Rad) and membranes were blocked for 1 h in 5% (w/v) non-fat milk in ddH_2O at room temperature (RT). Membranes were incubated at 7°C overnight with primary anti-spike antibody (43A11, rat IgG, Helmholtz Zentrum München; 1A9, mouse IgG, GeneTex; PA5-81795, polyclonal rabbit antibody, Invitrogen) at 1:2000 dilution in 5% (w/v) non-fat milk (Carl Roth) in ddH_2O, washed 3 times in TBST (Tris-buffered saline with 0.1% Tween-20) and incubated at RT for 1 h with horseradish peroxidase (HRP)-

conjugated secondary antibody (1112-035-062, goat anti-rat IgG, Jackson Immuno Research Europe; 7076S, horse anti-mouse IgG, Cell Signaling; 7074S, goat anti rabbit IgG, Cell Signaling) at 1:20,000, 1:2000 and 1:2000 dilution respectively, in 5% (w/v) non-fat milk in PBST (PBS, 0.05% Tween-20). After repeated (3×) washing in TBST, blots were incubated with ECL reagent (GE Healthcare) and imaged using a Fusion FX (Vilber).

ELISA

For the quantitation of spike in samples from various sources, a sandwich enzyme-linked immunosorbent assay (ELISA) was developed, using two anti-spike antibodies with non-overlapping epitopes. First, wells of a Nunc MaxiSorp plate (Thermo Fisher Scientific) were coated at RT for 5 h with 2 µg mL^{-1} anti-spike capture antibody (55E10, rat IgG, Helmholtz Zentrum München) or isotype in PBS. After washing with PBST (PBS, 0.05% Tween-20), free binding sites were blocked at RT for 2 h in 5% (w/v) non-fat milk (Carl Roth) in PBS. Samples of recombinant spike protein (S1+S2 extracellular domain, 40589-V08B1, Sino Biological), S^+ VLPs, or controls were diluted as indicated (Fig. 3A, B) in PBS and incubated at 7°C for 16 h on the antibody coated plate. After washing, HRP conjugated anti-spike detection antibody (43A11, rat IgG, Helmholtz Zentrum München) diluted 1:500 in 5% (w/v) non-fat milk in PBS) was added at RT for 2 h, washed again and incubated at RT with 100 µL TMB substrate reagent (BD555214, Becton Dickinson). The reaction was stopped by adding 50 µL 1 M H_2SO_4 and absorbance was measured at 450 nm in a CLARIOstar Plus (BMG Labtech). Data analysis was performed with GraphPad Prism 9.2.

NTA Analysis

Nanoparticle tracking analysis (NTA) was performed with the ZetaView PMX110 instrument (Particle Metrix) and the corresponding software (ZetaView 8.04.02) was used to measure the number and the size distribution of the S^+ VLP, virus stock and EV preparations. Samples were diluted in filtered PBS to achieve a vesicle concentration of approximately 1×10^7 mL^{-1}. Pre-acquisition parameters were set to a sensitivity of 75, a shutter speed of 50, a frame rate of 30 frames per second and a trace length of 15. The post-acquisition parameters were set to a minimum brightness of 20, a minimum size of 5 pixels and a maximum size of 1000 pixels.

Analysis of S^+ VLP, SARS-CoV-2 and EV particles with nano flow technology

Single particles were analyzed using our nano flow technology and a CytoFLEX LX cytometer (Beckman Coulter Life Science) with samples of S^+ VLPs, SARS-CoV-2 stock and EVs pre-stained with CellTraceViolet (CTV, Thermo Fisher Scientific) at 37°C for 20 min and quenched with PBS with 1% BSA. Upon washing via a 100 kDa cutoff Amicon ultra filter, the samples were stained with AlexaFluor488 conjugated anti-S antibody (43A11, rat IgG, Helmholtz Zentrum München), diluted and afterwards analyzed in the flow cytometer.

Cryo-electron microscopy

Aliquots of 4 µL from crude or purified S^+ VLPs were deposited on EM grids coated with a perforated carbon film. After draining the excess liquid with a filter paper, grids were quickly plunged into liquid ethane cooled by liquid nitrogen, using a Leica EMCPC cryo-chamber, and stored in cryo-boxes under liquid nitrogen until use. For cryo-EM observation, grids were mounted onto a Gatan 626 cryoholder and transferred to a Tecnai F20 microscope (FEI, USA) operated at 200 kV. Images were recorded with an Eagle 2k CCD camera (Thermo Fisher, USA).

EV fusion assay with BlaM readout

For EV fusion or EV neutralization assays with cytometric readouts, 2×10^4 Vero, $hACE2^+$ or U251MG, $hACE2^+$ cells (CID4608, CID4663, Helmholtz Zentrum München) in 100 µL DMEM (8% FBS, Pen/Strep supplemented) were seeded per well in a 96-well plate (353072, Falcon) and incubated o/n at 37°C, 5% CO_2. Where indicated, the cells were pre-incubated at 37°C for 1 h with chloroquine (C6628, Sigma-Aldrich, chloroquine diphosphate dissolved in ddH_2O) or were left without further treatment. The spent medium was replaced with up to 100 µL concentrated EVs (S^+, mock or VSV-G^+, CD63~$BlaM^+$) with or without prior pre-incubation with serum samples. After 4 h incubation at 37°C, 5% CO_2, the supernatant was removed, adherent cells were washed in PBS, trypsinized and moved to a V-bottom shaped 96-well plate and sedimented by centrifugation. After washing the cells once in CO_2 independent medium (8% FBS, Pen/Strep supplemented, Gibco, Thermo Fisher Scientific), 50 µL staining solution were added and the cells were incubated at RT in the dark o/n. The staining solution was composed of 2 µL CCF4-AM, 8 µL solution B (K1095, Thermo Fisher Scientific) and 10 µL 250 mM Probenecid (P8761, Sigma-Aldrich) per 1 mL supplemented CO_2 independent medium. After enzymatic turnover of the CCF4, cells were washed once with PBS (+3% FBS) and analyzed by flow cytometry using an LSR Fortessa instrument (BD) with a high throughput auto sampler. The 409-nm wavelength laser (violet) was used for excitation of the FRET substrate, resulting in emission of intact, non-cleaved CCF4 substrate at 520 nm (green), whereas emission of cleaved CCF4 substrate was detected at 447 nm (blue).

VLPNT with nanoLuciferase readout

For virus-like-particle neuralization tests (VLPNTs), 2×10^4 U251MG ($hACE2^+$, NM~$LgBiT^+$) cells (CID4697, Helmholtz Zentrum München) in 100 µL DMEM (8% FBS, Pen/Strep supplemented) were seeded per well in a 96-well Lumitrac200 (655075, Greiner Bio-One) plate and incubated at 37°C, 5% CO_2 overnight. The following day, serial dilutions of serum samples by a factor of 2 starting at 1:2 to 1:360 were prepared in PBS in a 96-well plate. 12 µL of the serial serum dilutions each were added to 48 µL normalized S^+ VLP (S, M, N, E, CD63~HiBiT) to obtain a series of final dilutions ranging from 1:10 to 1:1800. S^+ VLPs and serum dilutions were incubated at 37°C for 30 min in a 96-well plate. Thereafter, 50 µL was transferred to recipient cells after removal of their culture medium and the cells were incubated at 37°C, 5% CO_2 for 4 h. Prior to readout, the supernatant was removed and replaced with 25 µL substrate mix (20 µL OptiMEM, Gibco, Thermo Fisher Scientific +5 µL nano-Glo diluted 1:20 in LCS buffer, N2012, Promega). Bioluminescence was immediately

quantified in a CLARIOstar Plus reader (BMG Labtech). Mean luminescence level of S^+ VLPs, only, was set to 0% neutralization; while background luminescence obtained with ΔvFP EVs was set to 100% neutralization. For serum or antibody samples, the titers that corresponded to 50% neutralization were determined by modeling nonlinear, sigmoidal 4PL curves using Graphpad Prism 9.3.1 and calculating values that correspond to 50% signal reduction after background correction. This value was termed $VLPN_{50}$. The WHO international standard (NIBSC 20/136) was used in in-between runs and as within-run reference. For inhibitor experiments, cells were pre-incubated for 1 h at 37°C in 100 µL DMEM (8% FBS, Pen/Strep supplemented) with chloroquine (C6628, Sigma-Aldrich, chloroquine diphosphate dissolved in ddH_2O) or camostat-mesylate (SML0057, Sigma-Aldrich, dissolved in DMSO) prior to incubation with S^+ VLPs.

Statistical analysis

Data and statistical analyses were performed using Prism 9.3.1 (GraphPad Software, California, USA). For main-, as well as supplementary figures, *arithmetic* mean values are displayed. Error bars for y-axes indicate standard deviation (SD) of at least three replicates or more if indicated. Prior to t-tests and one-way ANOVA, normal- or log_{10}-normal distribution according to Gaussian distribution was assured.

Anti-spike antibody generation

To obtain spike antigen in its authentic conformation for the immunization of rats, a constitutive spike expressing HEK293 cell line (CID4618, Helmholtz Zentrum München) expressing SARS-CoV-2 $spike^{FL}$ (D614G) was established. To boost expression levels further, the cells were transiently transfected with an S expression plasmid (p7413, Helmholtz Zentrum München) prior to the isolation of S^+ EVs from conditioned media three days after transfection. EVs were purified and concentrated by ultrafiltration, density gradient ultra-centrifugation and subsequently characterized as described above. For the generation of anti-spike antibodies, S^+ EVs were used to immunize rats in a prime-boost-boost scheme, before splenic B-cells were isolated to establish hybridoma cell clones. Specific clones were subsequently identified by screening of hybridoma supernatants for binding to the constitutive spike expressing HEK293 cell line (CID4618, Helmholtz Zentrum München) via flow cytometry. The resulting anti-spike antibodies (COVEV: 43A11, 55E10 and 35B12) were then characterized thoroughly to validate their affinities and specificities for spike.

Antibody production

Antibodies 43A11, 55E10 and 35B12 were purified from hybridoma supernatants (Helmholtz Zentrum München Core Facility Monoclonal Antibodies). Antibodies REGN10987 and REGN10933 were purified from CHO cells transiently transfected with expression plasmids encoding the heavy and light chains. Briefly, cell culture supernatant was loaded on a protein GammaBind Plus Sepharose (17088602, Cytiva) or HiTrap MabSelect SuRe (29049104, Cytiva) column, washed with PBS and antibodies were eluted at pH 2.7 or 3.5, respectively in citric acid buffer. Fractions were pooled, neutralized with 1 M Tris (pH 8.0) and separated on Superdex200 prep grade (17104301, Cytiva) in PBS for monomers or buffer exchanged to PBS via ultra-

filtration, respectively. Directly coupled AlexaFluor488 labelled antibodies were generated using commercial kits (A10235, Thermo Fisher Scientific).

SARS-CoV-2 pseudotyped virus neutralization test (pVNT)

S-pseudotyped retrovirus stocks were generated by co-transfecting expression plasmids coding for S, gag and pol (from murine leukemia virus, MLV) and a myeloproliferative sarcoma virus (MPSV) based retroviral vector encoding eGFP (p7413, p4037, p6895, Helmholtz Zentrum München) into HEK293T cells. After 48 h, the supernatant was harvested and centrifuged at 7°C for 10 min at 300×g, 20 min at 4200×g and flash frozen for storage (-80°C). For pVNTs, 5×10^3 Vero ($hACE2^+$) cells (CID4608, Helmholtz Zentrum München) in 100 µL DMEM (8% FBS, Pen/Strep supplemented) were seeded per well in a 96-well plate (353072, Falcon) and incubated at 37°C, 5% CO_2 overnight. The cells were then pre-incubated for 1 h at 37°C in 50 µL, 8 µg mL^{-1} protamine sulfate in DMEM with or without chloroquine (C6628, Sigma-Aldrich, chloroquine diphosphate dissolved in ddH_2O) and retroviral stocks were pre-incubated for 30 min at 37°C with 5× serum dilutions in PBS. 50 µL of this mix where then added to the pre-incubated cells and centrifuged for 90 min at 800×g, at 37°C. After 48 h at 37°C, GFP expression was quantified by flow cytometry using an LSR Fortessa instrument (BD).

SARS-CoV-2 neutralization test (VNT_{100})

Human sera were heat-inactivated at 56°C for 30 min and diluted in a series of two-fold dilution steps (1:4 to 1:512). 100 plaque-forming units (PFU) of SARS-CoV-2 stock (German isolate BavPat1/2020; European Virus Archive Global # 026 V-03883, GenBank: MZ558051.1) contained in 50µL was added to an equal volume of diluted serum. The mixture was incubated at 37°C and approximately 20,000 Vero C1008 cells (ATCC, Cat#CRL-1586) were added after 1 hour. After a four-day incubation period, the cytopathic effect (CPE) was evaluated by light microscopy. Virus neutralization was defined as the complete absence of CPE in a given serum dilution (VNT_{100}). The reciprocal geometric mean titer (GMT) was calculated from the highest serum dilution without CPE based on three replicates. The lower detection limit of the assay was 1:8, corresponding to the first dilution of the serum tested. Two positive controls were used as inter-assay neutralization standards and quality control for each test. The WHO international standard (NIBSC 20/136) was tested seven times, resulting in a GMT of 377 for 100% absence of CPE in this test. Neutralization assays were performed in the BSL-4 laboratory of the Institute of Virology at Philipps University Marburg, Germany (1).

Patients and specimens

We included 63 serum specimens collected between April 6, 2020, and December 29, 2020, from 40 patients infected with SARS-CoV-2 at the LMU Klinikum, Munich, Germany. Patients are part of the COVID-19 Registry of the LMU Klinikum (CORKUM, WHO trial id DRKS00021225) and the study was approved on March 23, 2020 by the ethics committee (no. 20-245) of the Faculty of Medicine of the LMU (Ethik-Kommission bei der Medizinischen Fakultät der Ludwig-Maximilians-Universität München, Pettenkoferstr.

8a, 80336 München, Germany). Clinical data were obtained from health records and all patient data were anonymized for analysis. All patients were tested positive for SARS-CoV-2 by rRT-PCR in nasopharyngeal or oropharyngeal swabs. The median age of the 40 COVID-19 patients was 60 years (interquartile range 52 to 72 years), and 27.5% (11/40) of these individuals were female. We categorized the disease severity of the COVID-19 patients according to the WHO guideline "Clinical Management of COVID-19" (2): asymptomatic (no clinical signs of infection), mild (symptomatic patients without evidence of viral pneumonia or hypoxia), moderate (clinical signs of pneumonia, including fever, cough, dyspnoea), severe (clinical signs of pneumonia, plus one of the following: respiratory rate >30 min^{-1}, severe respiratory distress, SpO_2 <90% on room air), critical (one of the following: acute respiratory distress syndrome, sepsis, septic shock). Three patients were categorized as asymptomatic, 4 patients as mild, 11 patients as moderate, 11 patients as severe, and 11 patients as critical.

Virus stock preparation

CaCo-2 cells (American Type Culture Collection, ATCC, Virginia, USA) in cell culture medium (Dulbecco's Modified Eagle's Medium containing 2% FBS) were challenged for 2 h with a clinical isolate of SARS-CoV-2 (GISAID EPI ISL 2967222) previously obtained from a nasopharyngeal swab of a COVID-19 patient. Subsequently, cell culture medium was exchanged, and three days post infection supernatants were passaged on Vero-E6 cells (ATCC, Virginia, USA). After three additional days, the cell culture supernatant was harvested and stored at -80°C. The virus stock was characterized by rRT-PCR. Heat-inactivation of the virus stock was performed by incubating the sample for 30 min at 56°C.

References

1. A. J. Romero-Olmedo *et al.*, Induction of robust cellular and humoral immunity against SARS-CoV-2 after a third dose of BNT162b2 vaccine in previously unresponsive older adults. *Nat Microbiol* **7**, 195-199 (2022).

2. World Health Organization (2020) Clinical management of COVID-19: interim guidance, 27 May 2020. (World Health Organization, Geneva).

Supporting Figures

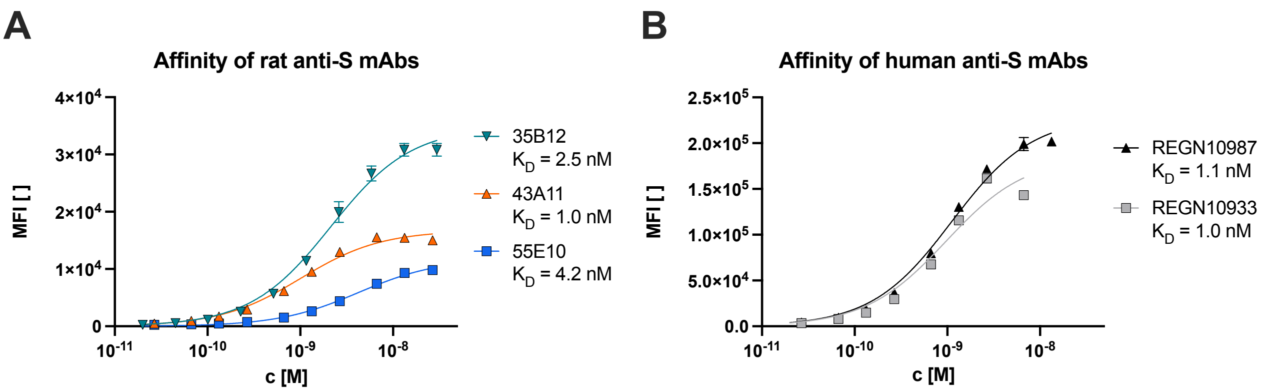

Supplementary Figure S1. Binding and dissociation constant estimation of five spike-specific mAbs

Binding affinities of five recombinant anti-spike mAbs were analyzed by flow cytometry using a HEK293 cell line that expresses spike protein B.1 (S:Wuhan-2019, D614G) constitutively. Mean fluorescence intensity (MFI) values were determined for various concentrations (c) of the antibodies with the aid of a labeled secondary antibody. MFI data of biological triplicates were analyzed according to a one-site specific binding model to estimate the dissociation constant K_D of the mAb to its antigen.

(A) Binding curves of three in house generated rat IgG anti-S mAbs 35B12, 43A11 and 55E10 are shown.

(B) Binding curves of two spike-specific neutralizing human IgG mAbs, REGN10987 and REGN10933, are shown for comparison.

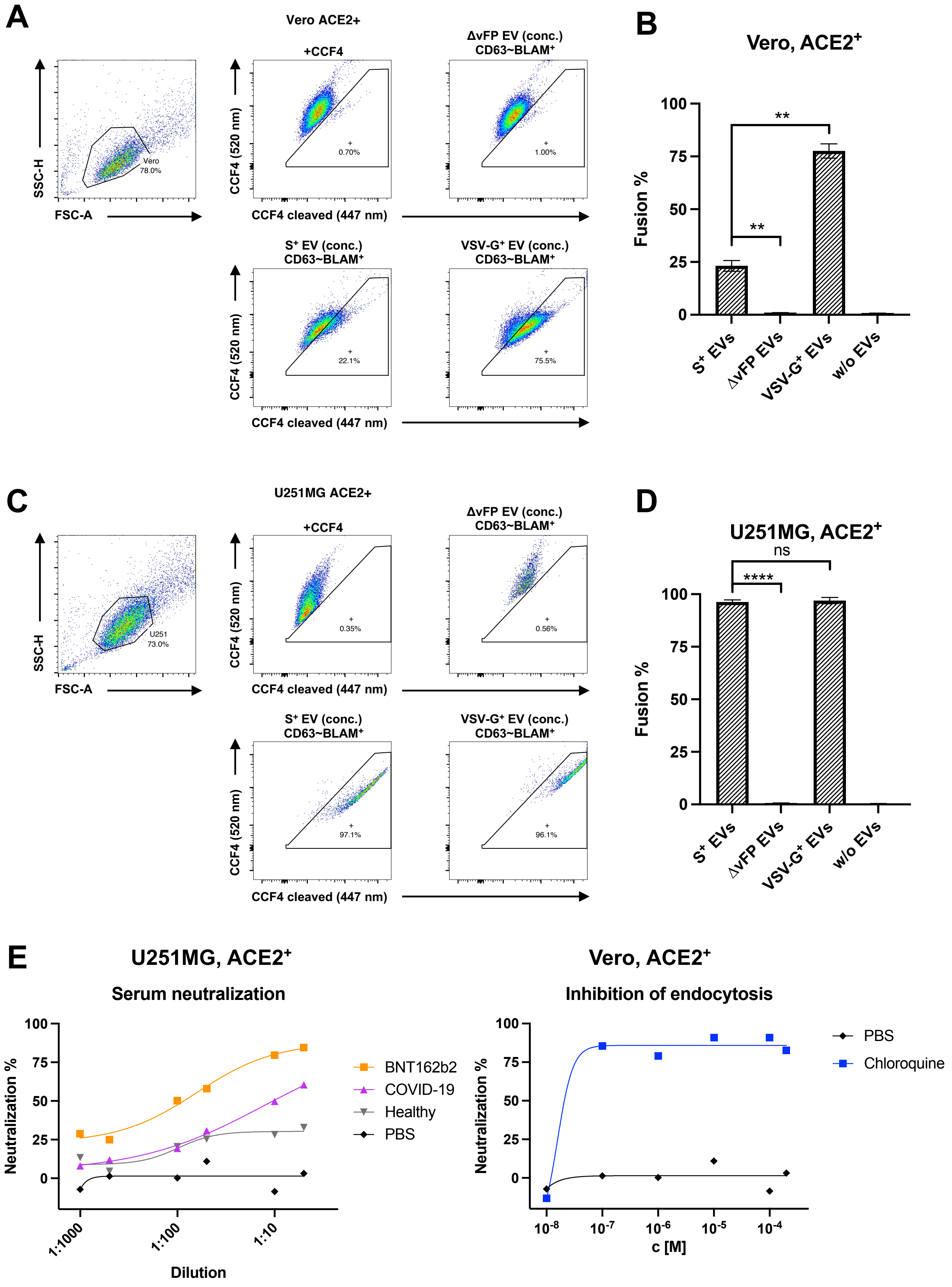

Supplementary Figure S2. VLPNT with the BlaM reporter system

A version of the spike-specific VLPNT is shown which uses extracellular vesicles (EVs) and β-lactamase (BlaM) as protein reporter. It consists of a fusion of BlaM to the carboxy-terminus of human CD63, which is efficiently incorporated into the membrane of EVs. HEK293T cells transiently co-transfected with

CD63~BlaM and spike encoding plasmids release extracellular vesicles termed S^+ EVs. Upon incubation of $ACE2^+$ recipient cells, CD63~BlaM is transferred to the cells' cytoplasm and BlaM cleaves the substrate CCF4 altering the emission spectrum of the cells, which is analyzed by flow cytometry. BlaM-positive cells within the gate show blueish fluorescence (x-axis) compared with greenish cells, which contain uncleaved CCF4 (y-axis).

(A) Flow cytometry plots show the gating strategy of intact Vero cells (left panel) as recipient cells, engineered to express human ACE2 and loaded with the BlaM substrate CCF4. The top row middle and right panels show untreated Vero $hACE2^+$ cells and cells incubated with concentrated (conc.) ΔvFP EVs, respectively. The ΔvFP EVs contain CD63~BlaM, only, but lack the viral fusion protein (vFP). The two bottom panels show examples of recipient Vero $hACE2^+$ cells after the successful delivery of ß-lactamase using two different types of particles. The lower left and right panels show Vero $ACE2^+$ cells incubated with S^+ EVs and VSV-G^+ EVs, respectively. The fraction of cells which have taken up VSV-G^+ EVs is larger compared to the fraction of cells which were incubated with S^+ EVs.

(B) Data from four biological replicates (examples are shown in panel A) are summarized and graphically displayed. Results from t-tests are indicated; ns, not significant ($p > 0.05$); $^*p \leq 0.05$; $^{**}p \leq 0.01$; $^{***}p \leq 0.001$; $^{****}p \leq 0.0001$.

(C, D) Experiments were conducted as in panels A and B with U251MG recipient cells stably expressing human ACE2. They were found to be better targets for S^+ EVs than $ACE2^+$ Vero cells as 97% of the $ACE2^+$ U251MG cells incubated with S^+ EV contained cleaved CCF4 compared to 21.4% of $ACE2^+$ Vero cells in panels A and B.

(E) Neutralization experiments with three sera and a PBS control using $ACE2^+$ U251MG cells as recipients and S^+ EVs containing the CD63~BlaM protein reporter. In the left panel, concentration dependent neutralization of S^+ EVs using sera from an individual vaccinated with BNT162b2 and a COVID-19 convalescent are compared with a naïve, healthy donor (and PBS as negative control). Fusion of S^+ EVs with $ACE2^+$ Vero cells was efficiently blocked by low concentrations (c) of chloroquine indicative of the endosomal uptake of S^+ EVs (right panel).

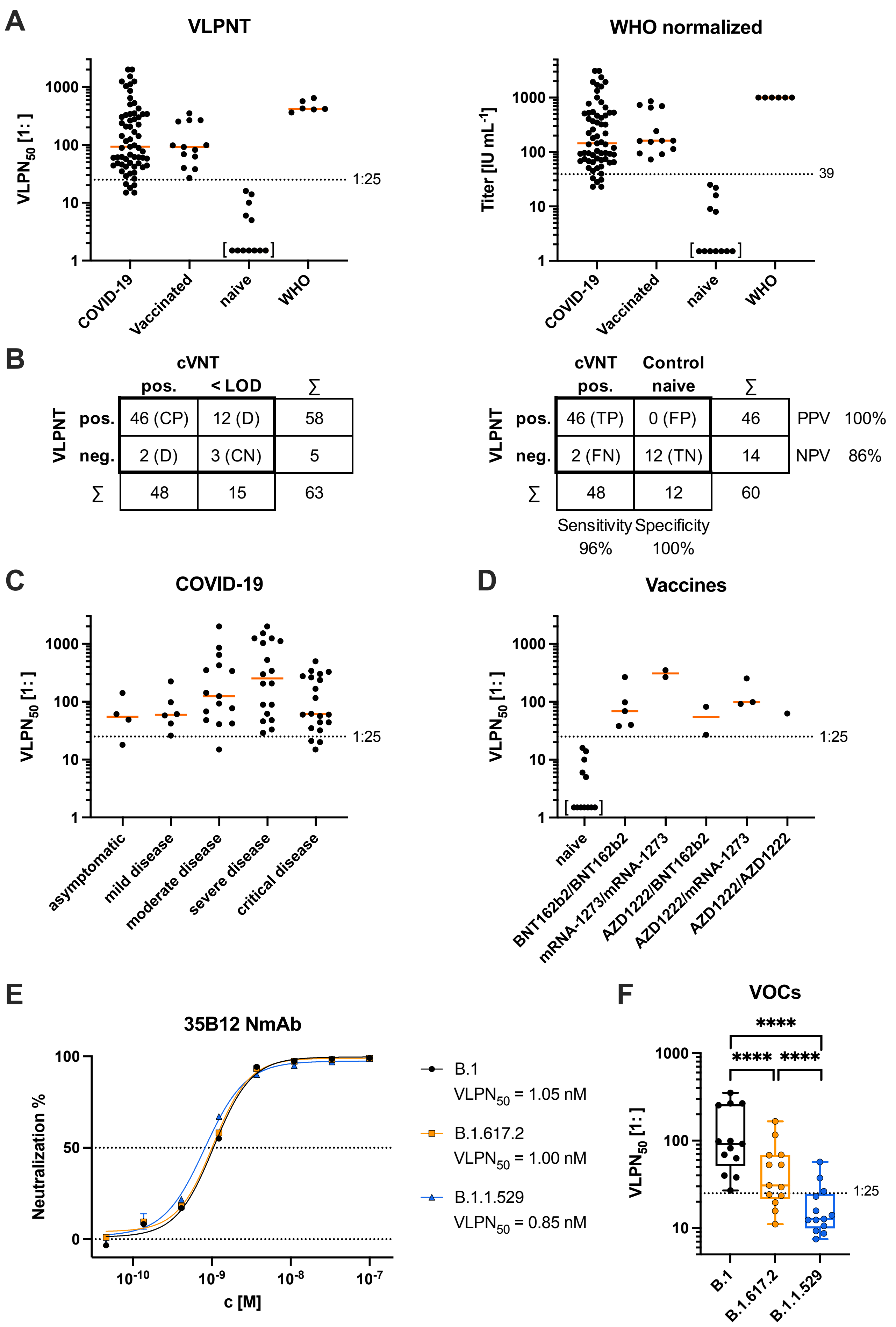

A
VLPNT
WHO normalized
VLPN50 [1:]
Titer [IU mL-1]
1:25
39
COVID-19
Vaccinated
naive
WHO
B
cVNT
pos.
< LOD
∑
VLPNT
pos.
46 (CP)
12 (D)
58
neg.
2 (D)
3 (CN)
5
48
15
63
cVNT
Control
pos.
naive
46 (TP)
0 (FP)
46
PPV 100%
2 (FN)
12 (TN)
14
NPV 86%
48
12
60
Sensitivity Specificity
96% 100%
C
COVID-19
asymptomatic
mild disease
moderate disease
severe disease
critical disease
D
Vaccines
naive
BNT162b2/BNT162b2
mRNA-1273/mRNA-1273
AZD1222/BNT162b2
AZD1222/mRNA-1273
AZD1222/AZD1222
E
35B12 NmAb
Neutralization %
c [M]
B.1
VLPN50 = 1.05 nM
B.1.617.2
VLPN50 = 1.00 nM
B.1.1.529
VLPN50 = 0.85 nM
F
VOCs
****
**** ****
B.1
B.1.617.2
B.1.1.529

Supplementary Figure S3. Diagnostic VLPNT performance and serum titer classification according to the donors' medical history.

(A) $VLPN_{50}$ titers of 63 sera from COVID-19 patients, 13 COVID-19 vaccinees, 12 healthy naïve donors and six independent replicates of the WHO convalescent COVID-19 reference serum (NIBSC 20/136) are grouped and shown with median values (orange bars). The dashed horizontal line indicates $VLPN_{50}$ titers below which the test does not score spike-specific neutralizing antibodies according to our criteria. The left and right panels show the grouped $VLPN_{50}$ titers prior to and after their normalization to international units (IU) with the WHO standard, respectively. Values in square brackets indicate sera with $VLPN_{50}$ values below the limit of detection (LOD).

(B) The left panel compares the results of 63 COVID-19 samples tested in the VLPNT and the cVNT. Concordant positive (CP), concordant negative (CN), and discrepant (D) results are indicated. In the right panel, sensitivity, specificity, positive predictive value (PPV) and negative predictive value (NPV) of the VLPNT were calculated based on the 48 COVID-19 serum samples with neutralizing capacity in the cVNT and 12 serum controls of healthy naïve donors collected prior to mid 2019. Test results are indicated as: pos., positive; neg., negative; < LOD, below limit of detection; TP, true positive; FN, false negative; FP, false positive; TN, true negative.

(C) $VLPN_{50}$ titers of 63 COVID-19 sera are sorted according to their patient's clinical disease course. Orange bars indicate median values of grouped data.

(D) $VLPN_{50}$ serum titers of vaccinees are shown according to their prime-boost scheme of different COVID-19 vaccines received. Orange bars indicate median values of the groups. Values in square brackets indicate serum samples with $VLPN_{50}$ below the LOD.

(E) Mean neutralization values of three biological replicates are displayed (error bars indicate standard deviation) after inter-batch and inter-VOC normalization based on adjusted relative light units. S^+ VLP preparations were analyzed using the SARS-CoV-2 pan-variant neutralizing monoclonal antibody (NmAb) 35B12. Almost congruent neutralization graphs demonstrate the efficient normalization of VLP preparations, which is further supported by three very close $VLPN_{50}$ values.

(F) Similar to Fig. 7C, $VLPN_{50}$ results of sera from 13 COVID-19 vaccinees are shown individually and as box plots together with mean neutralization values for SARS-CoV-2 VOCs B.1, B.1.617.2 and B.1.1.529 (BA.1). Data were analyzed using a matched one-way ANOVA, with Tukey's multi comparison test and a single pooled variance. Results are indicated: ****$p \leq 0.0001$.

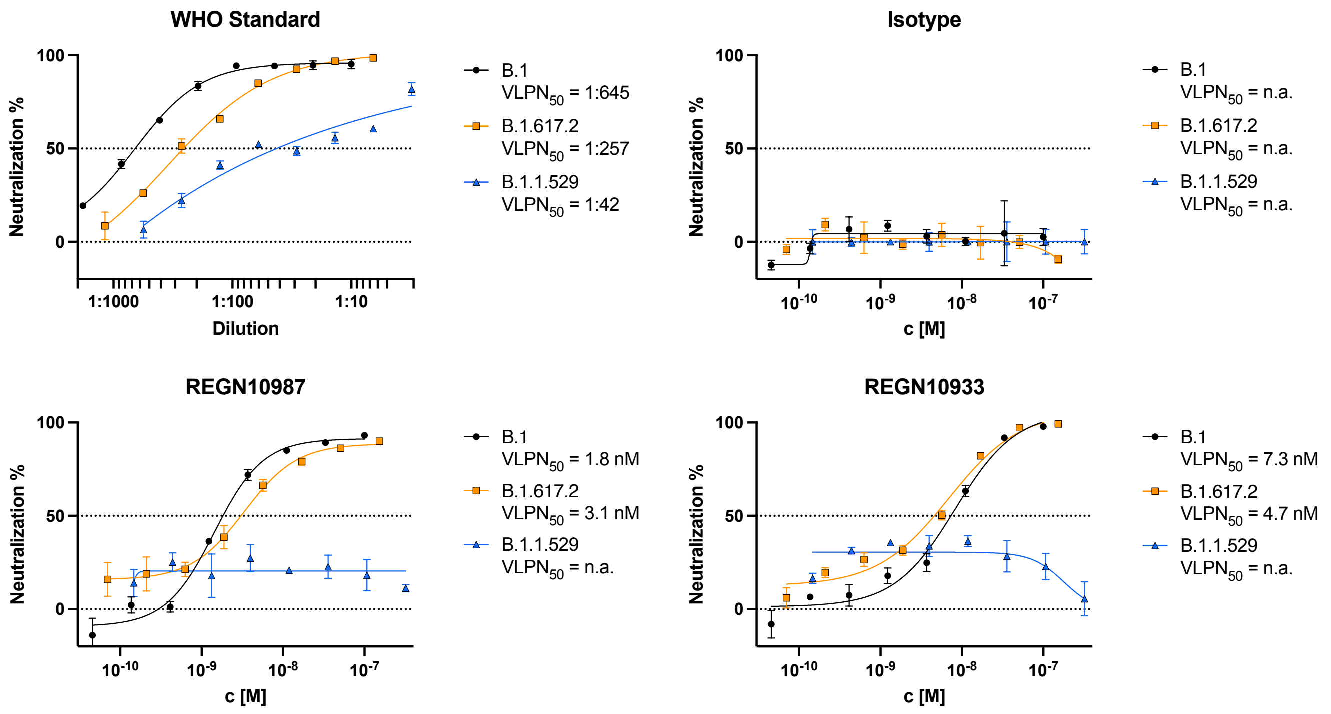

Supplementary Figure S4. Selection of VLPNT graphs and $VLPN_{50}$ values comparing three SARS-CoV-2 VOCs

The WHO reference serum (NIBSC 20/136) intended for international standardization and two recombinant human mAbs, REGN10987 and REGN10933, were analyzed for their neutralization capacity directed against B.1 (S:Wuhan-2019, D614G), B.1.617.2 (Delta-VOC) and B.1.1.529 (BA.1, Omicron-VOC) S^+ VLPs.

The WHO reference serum showed a successive reduction of neutralization titer following the evolution of SARS-CoV-2 VOCs: B.1 > B.1.617.2 > B.1.1.529. While the REGN mAbs displayed potent neutralization in the assay at low molar concentration (c) for B.1 and B.1.617.2, they failed to efficiently neutralize B.1.1.529. An IgG isotype control mAb did not show any neutralizing activity and served as negative control. Mean values of three biological replicates are displayed with error bars indicating standard deviation (SD).

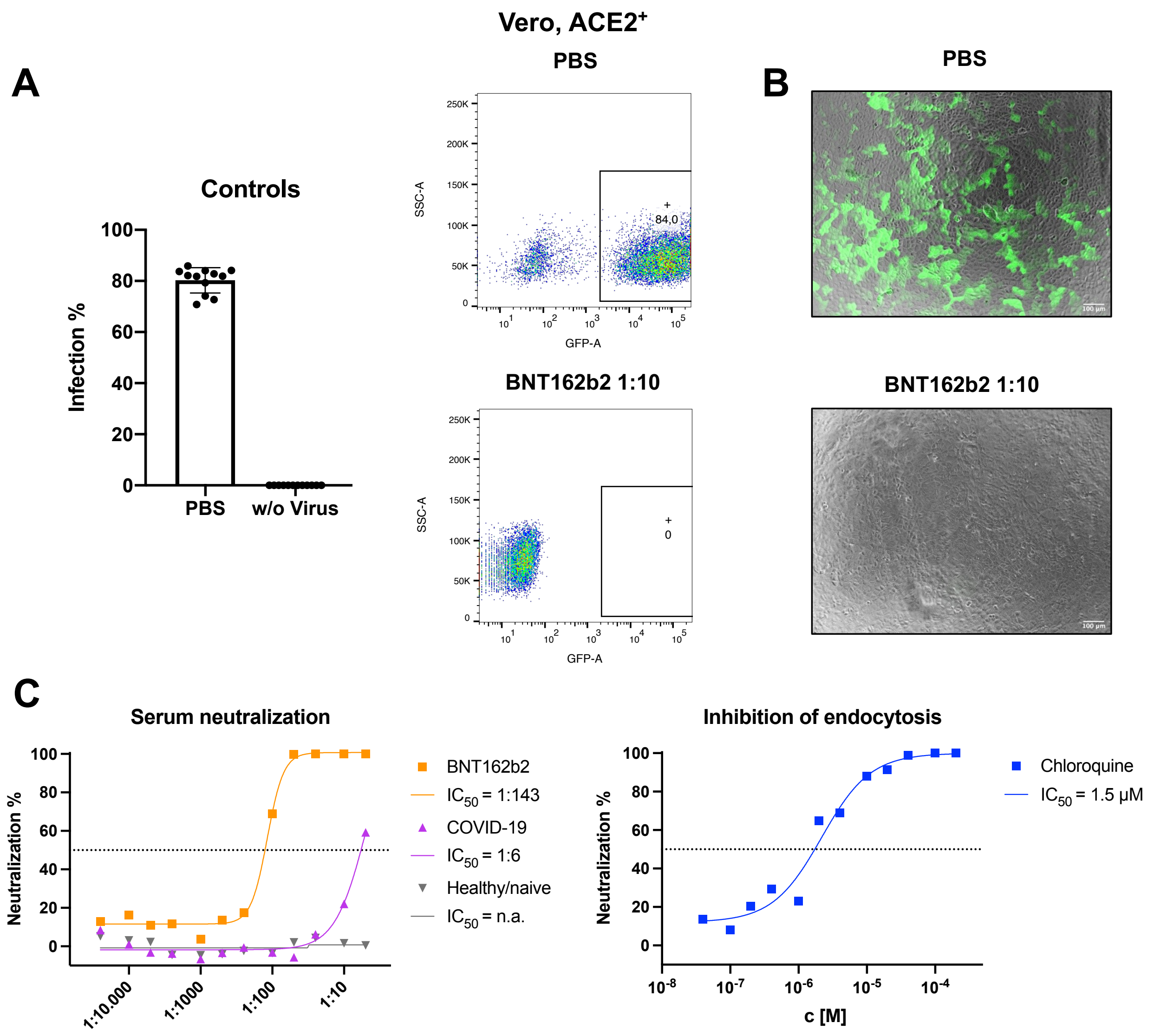

Supplementary Figure S5. pVNT with spike-pseudotyped retroviral vectors encoding GFP

Spike-pseudotyped retrovirus stock based on a murine leukemia virus vector encoding GFP was used in pseudotyped virus neutralization tests (pVNT) for the quantification of NAbs. Readouts rely on *de novo* expression of the retrovirally transduced GFP gene in $ACE2^+$ recipient cells analyzed by flow cytometry.

(A) A spike-pseudotyped retroviral vector preparation was incubated with Vero cells engineered to express human ACE2. The cells of 12 biological replicates were analyzed for GFP expression 48 h post infection by flow-cytometry yielding about 80% GFP^+ cells (top dot plot). Preincubation of the retroviral vector inoculum with a final concentration of a 1:10 diluted serum from a BNT162b2 vaccinated individual for 30 min efficiently blocked GFP expression (bottom plot).

(B) Merged fluorescence and brightfield microscopic images of Vero $ACE2^+$ cells visualize recipient cells infected with spike-pseudotyped retroviral vectors incubated with PBS as a negative control (top) and after adding neutralizing serum (bottom) as examples of the experiment described in panel A. White scale bar corresponds to 100 μm in length.

(C) Three sera from a vaccinee after BNT162b2 prime-boost immunization, a COVID-19 convalescent and a healthy naïve donor were titrated with fixed doses of spike-pseudotyped retroviral vector inoculum (left panel).

Infection of $ACE2^+$ Vero cells with pseudotyped vector particles was efficiently blocked by pre-incubating recipient cells with low concentrations (c) of chloroquine preventing endocytosis (right panel). Half maximal inhibitory concentrations IC_{50} are calculated, or indicated as not applicable (n.a.).

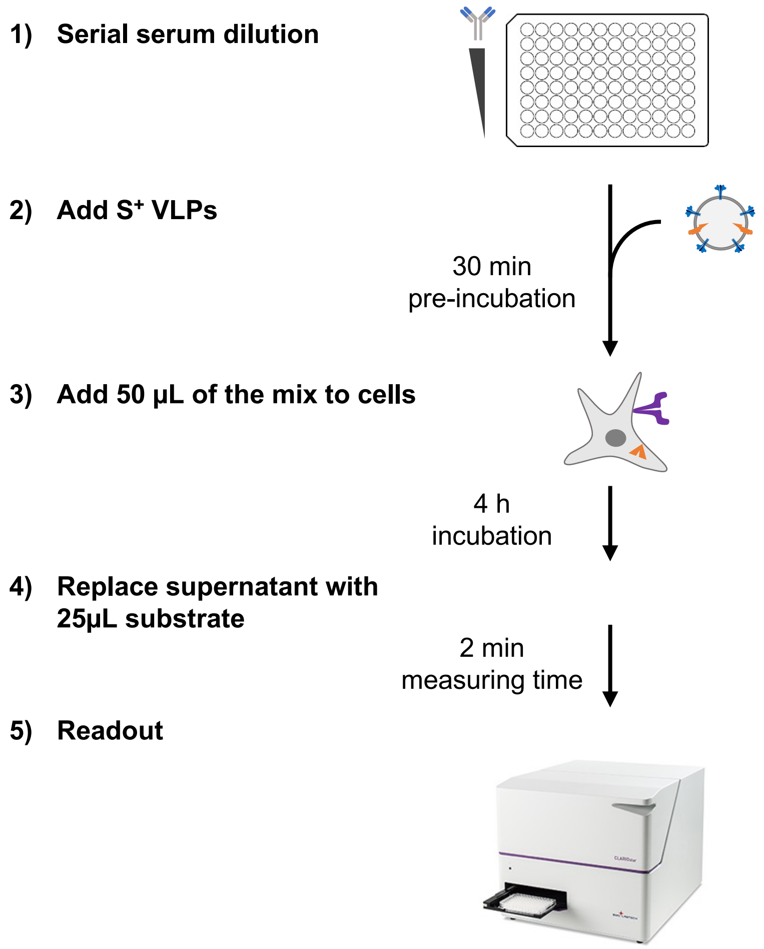

Supplementary Figure S6. Workflow of individual steps of the VLPNT

Serial dilutions of serum samples are prepared in a 96-well plate and S^+ VLPs are added followed by a pre-incubation step at 37°C for 30 min. 50 µL of the serum-VLP mix is added to previously seeded adherent target cells in a 96-well plate and incubated at 37°C for 4 h. The supernatant is removed and replaced with 25 µL of the luciferase substrate mix. After 2 minutes, the wells are analyzed for their bioluminescence in a standard luminometer.

Supplementary Table S1. Individual data of analyzed human serum samples.

											VLPNT				cVNT	
							Vaccination scheme				B.1		B.1.617.2	B.1.1.529	B.1	
	ID	Sex	Age	Sampling	COVID-19 severity	RT-qPCR	Prime	Boost	d p. PCR[a]	d p. Boost[b]	VLPN$_{50}$ [1:]	Titer$_{50}$[c] [IU/mL]	VLPN$_{50}$ [1:]	VLPN$_{50}$ [1:]	VNT$_{100}$[d] [1:]	Titer$_{100}$ [IU/mL]
COVID-19 patients	A01	f	31	Jun 2020	asymptomatic	+			104		49	76			8	21
	A02	f	62	Jun 2020	moderate dis.	+			54		352	546			81	215
	A03	m	60	Jun 2020	asymptomatic	+			79		18	28			8	21
	A04	m	32	Jun 2020	severe dis.	+			76		>2000	>3101			≥1024	≥2716
	A05	m	57	May 2020	mild dis.	+			17		26	40			†4	†11
	A06	m	36	May 2020	mild dis.	+			70		61	95			16	42
	A07	m	75	May 2020	severe dis.	+			4		89	138			†4	†11
	A08	m	51	May 2020	moderate dis.	+			57		>2000	>3101			≥1024	≥2716
	A09	m	84	May 2020	severe dis.	+			53		89	138			25	66
	A10	m	57	May 2020	mild dis.	+			11		58	90			†4	†11
	A11	m	58	May 2020	moderate dis.	+			38		340	527			102	271
	A12	m	73	May 2020	critical dis.	+			13		329	510			203	538
	B01	m	62	May 2020	asymptomatic	+			15		142	220			81	215
	B02	m	54	May 2020	moderate dis.	+			26		645	1000			≥1024	≥2716
	B03	m	86	Apr 2020	moderate dis.	+			9		426	660			323	857
	B04	f	31	May 2020	asymptomatic	+			54		61	95			†4	†11
	B05	m	84	Apr 2020	severe dis.	+			22		46	71			40	106
	B06	m	84	Apr 2020	severe dis.	+			18		29	45			10	27
	B07	m	59	Apr 2020	severe dis.	+			12		1527	2367			512	1358
	B08	m	88	Apr 2020	severe dis.	+			2		48	74			†4	†11
	B09	m	86	Apr 2020	critical dis.	+			2		44	68			†4	†11
	B10	m	59	Apr 2020	severe dis.	+			8		1250	1938			645	1711
	B11	m	29	Apr 2020	moderate dis.	+			1		125	194			81	215
	C01	f	71	Apr 2020	moderate dis.	+			17		143	222			161	427
	C02	f	69	Apr 2020	critical dis.	+			28		62	96			†4	†11
	C03	f	69	Apr 2020	critical dis.	+			30		60	93			†4	†11
	C04	f	31	Apr 2020	mild dis.	+			50		42	65			†4	†11
	C05	m	62	Apr 2020	critical dis.	+			15		21	33			8	21
	C06	m	36	May 2020	mild dis.	+			21		98	152			81	215
	C07	m	54	May 2020	moderate dis.	+			23		857	1329			≥1024	≥2716
	C08	f	67	May 2020	severe dis.	+			30		62	96			20	53
	C09	f	69	May 2020	critical dis.	+			48		20	31			†4	†11
	C10	m	56	May 2020	moderate dis.	+			44		42	65			10	27
	C11	m	59	Dec 2020	critical dis.	+			38		57	88			†4	†11
	C12	m	58	Dec 2020	critical dis.	+			15		15	23			†4	†11
	C13	m	77	Nov 2020	severe dis.	+			18		207	321			128	340
	C14	m	47	Dec 2020	severe dis.	+			15		1250	1938			≥1024	≥2716
	C15	f	59	Dec 2020	moderate dis.	+			49		41	64			8	21
	C16	m	88	May 2020	severe dis.	+			30		299	464			256	679
	C17	m	56	May 2020	moderate dis.	+			47		48	74			10	27
	C18	m	32	May 2020	severe dis.	+			14		33	51			†4	†11
	C19	m	43	May 2020	critical dis.	+			22		44	68			16	42
	C20	m	88	May 2020	severe dis.	+			37		341	529			256	679
	C21	m	56	May 2020	moderate dis.	+			51		68	105			10	27
	C22	m	56	May 2020	moderate dis.	+			54		77	119			16	42
	C23	m	32	May 2020	severe dis.	+			21		1035	1605			323	857
	C24	m	81	May 2020	critical dis.	+			50		341	529			406	1077
	C25	f	56	Dec 2020	mild dis.	+			43		224	347			102	271
	C26	m	58	Dec 2020	critical dis.	+			26		116	180			†4	†11
	C27	f	68	Dec 2020	moderate dis.	+			27		15	23			†4	†11
	C28	f	63	Jun 2020	critical dis.	+			18		60	93			20	53
	C29	f	72	Jun 2020	critical dis.	+			25		35	54			8	21
	C30	m	72	Jun 2020	critical dis.	+			43		500	775			20	53
	C31	m	43	Jun 2020	critical dis.	+			47		237	367			203	538
	C32	m	72	Jun 2020	critical dis.	+			47		303	470			16	42
	C33	m	23	Jun 2020	moderate dis.	+			49		93	144			51	135
	C34	m	43	Jun 2020	critical dis.	+			54		32	50			8	21
	C35	m	72	Jun 2020	critical dis.	+			54		263	408			10	27
	C36	m	43	Jun 2020	critical dis.	+			35		168	260			128	340
	C37	m	72	Jun 2020	critical dis.	+			35		276	428			25	66
	C38	m	43	Jul 2020	severe dis.	+			19		1112	1724			≥1024	≥2716
	C39	m	59	Nov 2020	severe dis.	+			14		206	319			81	215
	C40	m	76	Nov 2020	severe dis.	+			16		528	819			512	1358

Continued on next page

Group	ID	Sex	Age	Sampling	COVID-19 severity	RT-qPCR	Vaccination scheme: Prime	Vaccination scheme: Boost	d p. PCR[a]	d p. Boost[b]	VLPNT B.1 $VLPN_{50}$ [1:]	VLPNT B.1 $Titer_{50}$[c] [IU/mL]	VLPNT B.1.617.2 $VLPN_{50}$ [1:]	VLPNT B.1.1.529 $VLPN_{50}$ [1:]	cVNT B.1 VNT_{100}[d] [1:]	cVNT B.1 $Titer_{100}$ [IU/mL]
COVID-19 vaccinees	S001	f	93	Feb 2021			BNT162b2	BNT162b2		24	100	155	24	12		
	S005	m	26	May 2021			AZD1222	BNT162b2		13	82	113	31	9		
	S008	m	60	May 2021			AZD1222	BNT162b2		14	27	73	16	11		
	S011	f	28	Jul 2021			mRNA-1273	mRNA-1273		19	352	860	116	57		
	S012	m	66	Jul 2021			AZD1222	mRNA-1273		15	99	242	53	16		
	S014	m	70	Jul 2021			AZD1222	AZD1222		37	63	154	29	12		
	S015	f	32	Jul 2021			mRNA-1273	mRNA-1273		13	267	654	166	23		
	S016	f	59	Jul 2021			BNT162b2	BNT162b2		56	38	91	20	8		
	S025	f	68	Aug 2021			AZD1222	mRNA-1273		22	254	697	68	26		
	S026	m	66	Aug 2021			AZD1222	mRNA-1273		15	92	162	53	37		
	S027	f	58	Aug 2021			BNT162b2	BNT162b2		14	267	734	69	13		
	S033	f	60	Sep 2021			BNT162b2	BNT162b2		29	69	162	24	14		
	S034	m	59	Sep 2021			BNT162b2	BNT162b2		90	40	94	11	9		
Healthy, naive donors	S003	m	24	Aug 2019							5	8				
	S006	m	56	Oct 2017							14	22				
	S009	f	26	May 2019							†0	†0				
	S018	f	30	Oct 2017							16	25				
	S019	f	69	Nov 2017							†0	†0				
	S020	f	35	Nov 2017							†0	†0				
	S021	f	43	Dec 2017							†0	†0				
	S022	f	53	Dec 2017							†0	†0				
	S023	f	31	Oct 2017							†0	†0				
	S024	m	na	Dec 2017							6	9				
	S028	m	30	Mar 2019							†0	†0				
	S029	f	32	Mar 2019							10	16				
NIBSC 20/136	WHO	na	na	2020							645	*1000	257	42	377	*1000
	WHO	na	na	2020							409	*1000	422	43		
	WHO	na	na	2020							417	*1000				
	WHO	na	na	2020							364	*1000				
	WHO	na	na	2020							567	*1000				
	WHO	na	na	2020							426	*1000				

m, male; f, female; na, not available; dis., disease; d, days; p., post; *, per WHO definition; †, below limit of detection

[a]Days between first positive SARS-CoV-2 RT-qPCR report and sampling

[b]Days between booster dose and sampling

[c]$VLPN_{50}$ titer normalized to the WHO reference serum's within-run performance

[d]Reciprocal geometric mean titer (GMT) from the highest serum dilution displaying 100% reduction of CPE based on three replicates

List of Abbreviations

(+)ssRNA	Positive sense, single-stranded RNA
%	Percent
°C	Degree Celsius
ΔvFP	Without viral fusion protein
×g	Gravitational force equivalent
Ab	Antibody
ACE2	Angiotensin-converting enzyme 2
ADCC	Antibody-dependent cellular cytotoxicity
ADCP	Antibody-dependent cellular phagocytosis
ADE	Antibody dependent enhancement
ANOVA	Analysis of variance
ARDS	Acute respiratory distress syndrome
BlaM	β-lactamase
BSL	Biosafety level
BTI	Break-through infection
c	Concentration
CLSI	Clinical Laboratory Standards Institute
CMV	Cytomegalovirus
CN	Concordant negative
conc.	Concentrated
COVID-19	Coronavirus disease 2019
CP	Concordant positive
CPE	Cytopathic effect
ct	Threshold cycle
CTSL	Cathepsin L
CTV	CellTraceViolet (Thermo Fisher Scientific)
CV	Coefficient of variation
cVNT	Conventional virus neutralization test
D	Aspartic acid
D	Discrepant
Da	Dalton
dd	Double distilled
DMEM	Dulbecco's Modified Eagle's Medium
DMSO	Dimethyl sulfoxide
DNA	Deoxyribonucleic acid
E	Envelope protein
e.g.	*exempli gratia*
EBV	Epstein-Barr virus
ECD	Extracellular domain
ELISA	Enzyme-linked immunosorbent assay
EM	Electron microscopy
EMA	European Medicines Agency
EV	Extracellular vesicle
FBS	Fetal bovine serum
Fc	Fragment crystallizable region
FDA	U.S. Food and Drug Administration
FL	Full length
FN	False negative

FP	False positive
FP	Fusion protein
FRET	Fluorescence resonance energy transfer
FRNT	Focus reduction neutralization test
FSC	Forward scatter
g	Gram
G	Glycine
GFP	Green fluorescent protein
GMT	Geometric mean titer
h	Human
HIV	Human immunodeficiency viruses
HRP	Horseradish peroxidase
IC_{50}	Half maximal inhibitory concentration
IgG	Immunoglobulin G
IU	International units
k	kilo
kb	kilo base pair
KD	Dissociation constant
L	Liter
LOD	Limit of detection
log_{10}	Decadic logarithm
Luc	Luciferase
M	Membrane protein
m	Meter
m	milli
M	mol L^{-1}
mAb	Monoclonal antibody
MERS-CoV	Middle East respiratory syndrome Coronavirus
MFI	Mean fluorescence intensity
min	Minutes
MLV	Murine leukemia virus
MNA	Microneutralization assay
mol	Mole
MPSV	Myeloproliferative sarcoma virus
mRNA	Messenger RNA
N	Nucleoprotein
n	nano
n	Sample number
nLuc	nano-Luciferase
n.a.	Not available
na	Not available
NAb	Neutralizing antibody
NIBSC	UK National Institute for Biological Standards and Control
NM	*N*-myristoly
NmAb	Neutralizing monoclonal antibody
non-red.	Non-reducing
NPV	Negative predictive value
ns	Not significant
NTA	Nanoparticle tracking analysis
OD	Optical density
ORF	Open reading frames

p	p-value
PAGE	Polyacrylamide gel electrophoresis
PBS	Phosphate buffered saline
PBST	PBS, 0.05% Tween-20
PCR	Polymerase chain reaction
Pen	Penicillin
PFU	Plaque-forming units
PPV	Positive predictive value
PRNT	Plaque reduction neutralization test
$PRNT_{50}$	50% plaque reduction neutralization titer
pVNT	Pseudotyped virus neutralization test
r	Pearson correlation coefficient
RBD	Receptor binding domain
RBM	Receptor binding motif
red.	Reducing
REGN	Regeneron
RLU	Relative light units
RM	Repeated measures
RNA	Ribonucleic acid
rRT-PCR	Real-time reverse transcription PCR
RSV	Respiratory syncytial virus
RT	Room temperature
RT-qPCR	Reverse transcription quantitative real-time PCR
S	Spike protein
SARS-CoV	Severe acute respiratory syndrome Coronavirus
SARS-CoV-2	Severe acute respiratory syndrome Coronavirus 2
SD	Standard deviation
SDS	Sodium dodecyl sulfate
SpO2	Peripheral oxygen saturation
SSC-H	Side scatter height
Strep	Streptomycin
sVNT	Surrogate virus neutralization tests
TBS	Tris-buffered saline
TBST	Tris-buffered saline with 0.1% Tween-20
TMPRSS2	Transmembrane protease serine 2
TN	True negative
TP	True positive
Tris	Tris(hydroxymethyl)aminomethane
V	Volt
VLP	Virus-like-particle
$VLPN_{50}$	50% VLP neutralization titer
VLPNT	VLP neutralization test
VNT	Virus neutralization tests
VNT_{100}	100% virus neutralization titer
VNT_{50}	50% virus neutralization titer
VOC	Variant of concern
vRNA	Viral ribonucleic acid
VSV	Vesicular stomatitis virus
VSV-G	VSV glycoprotein G
w/o	Without
w/v	Weight per volume

WB	Western-blot
WHO	World Health Organization
WNV	West Nile virus
μ	micro
